# Robust and Adhesive Laminar Solid Electrolyte with Homogenous and Fast Li‐Ion Conduction for High‐Performance All‐Solid‐State Lithium Metal Battery

**DOI:** 10.1002/advs.202404307

**Published:** 2024-06-14

**Authors:** Shiyuan Guo, Yuefeng Su, Kang Yan, Chenying Zhao, Yun Lu, Haoyu Wang, Jinyang Dong, Ning Li, Yun Liu, Yibiao Guan, Feng Wu, Lai Chen

**Affiliations:** ^1^ School of Materials Science and Engineering Beijing Key Laboratory of Environmental Science and Engineering Beijing Institute of Technology Beijing 100081 P. R. China; ^2^ Chongqing Innovation Center Beijing Institute of Technology Chongqing 401120 P. R. China; ^3^ National Key Laboratory of Renewable Energy Grid‐Integration China Electric Power Research Institute Beijing 100192 P. R. China

**Keywords:** adhesion, all‐solid‐state lithium metal batteries, homogeneous and fast Li‐ion flux, mechanical modulus, thin laminar solid electrolytes

## Abstract

Constructing composite solid electrolytes (CSEs) integrating the merits of inorganic and organic components is a promising approach to developing high‐performance all‐solid‐state lithium metal batteries (ASSLMBs). CSEs are now capable of achieving homogeneous and fast Li‐ion flux, but how to escape the trade‐off between mechanical modulus and adhesion is still a challenge. Herein, a strategy to address this issue is proposed, that is, intercalating highly conductive, homogeneous, and viscous‐fluid ionic conductors into robust coordination laminar framework to construct laminar solid electrolyte with homogeneous and fast Li‐ion conduction (LSE‐HFC). A 9 µm‐thick LSH‐HFC, in which poly(ethylene oxide)/succinonitrile is adsorbed by coordination laminar framework with metal–organic framework nanosheets as building blocks, is used here as an example to determine the validity. The Li‐ion transfer mechanism is verified and works across the entire LSE‐HFC, which facilitates homogeneous Li‐ion flux and low migration energy barriers, endowing LSE‐HFC with high ionic conductivity of 5.62 × 10^−4^ S cm^−1^ and Li‐ion transference number of 0.78 at 25 °C. Combining the outstanding mechanical strength against punctures and the enhanced adhesion force with electrodes, LSE‐HFC harvests uniform Li plating/stripping behavior. These enable the realization of high‐energy‐density ASSLMBs with excellent cycling stability when being assembled as LiFePO_4_/Li and LiNi_0.6_Mn_0.2_Co_0.2_O_2_/Li cells.

## Graphical abstract

A thin laminar solid electrolyte can actualize the homogeneous and fast Li‐ion flux while also breaking the trade‐off between mechanical modulus and adhesion. The robust coordination laminar framework allows electrolytes to achieve a high Young's modulus against punctures. Viscous‐fluid ionic conductor confined in coordination laminar framework provides homogeneous and fast Li‐ion transport channels and adhesive contact with electrodes.

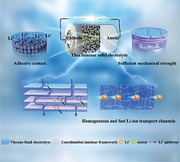

## 1 Introduction

Switching out liquid electrolytes with solid‐state electrolytes (SSEs) has been proven that the effective approach to overcome safety concerns of high‐energy‐density lithium metal batteries.^[^ ^1^, ^2^, ^3^ ^]^ To meet the requirement of all‐solid‐state lithium metal batteries (ASSLMBs) with satisfactory performance, SSEs should be equipped with the following merits. First, the homogeneous and fast Li‐ion flux in SSEs is imperative for suppressing the lithium dendrites growth induced by high local Li‐ion concentration.^[^ ^4^, ^5^ ^]^ Second, SSEs, acting as a compartment between the electrodes, ought to possess sufficient mechanical strength to prevent the short circuit during charge–discharge.^[^ ^6^ ^]^ Third, it is also indispensable to create the adhesive contact between SSEs and electrodes, which will enhance the Li‐ion transfer kinetics within the interfaces and further improve the cycling performance of the battery.^[^ ^7^, ^8^ ^]^ Unfortunately, the currently available SSEs, including inorganic solid electrolytes (ISEs), solid polymer electrolytes (SPEs), and composite solid electrolytes (CSEs), still cannot fully meet all the above requirements.

Specifically, ISEs (e.g., oxides and sulfides) generally exhibit a superior Li‐ion conductivity of 10^−4^ to 10^−2^ S cm^−1^ at room temperature (RT), but the Li‐ion flux within the ISEs is typically inhomogeneous, due to the significant difference in Li‐ion transfer impedance at grain boundaries and the grains.^[9−11]^ In addition, although the high mechanical modulus of ISEs helps inhibit the growth of lithium dendrites, it will also lead to undesirable electrolyte/electrode contact, which incurs the notoriously large interfacial resistance laying a giant gap for their practical application.^[^ ^12^ ^]^ Relatively, compared with ISEs, the softness of SPEs endows them with ameliorative interfacial contact.^[^ ^13^ ^]^ Besides, SPEs represented by poly(ethylene oxide) (PEO) electrolytes also feature lightweight, low‐cost, and superior film processability.^[^ ^14^, ^15^ ^]^ However, poor ionic conductivity and mechanical strength become stumbling blocks for SPEs in further application.^[^ ^16^ ^]^ For example, PEO electrolyte has only an ionic conductivity of 10^−7^ to 10^−6^ S cm^−1^ at RT arising from the high crystallinity of PEO chains and is also at risk of being punctured by lithium dendrites.^[^ ^17^ ^]^ In response to the hindrance, constructing CSEs by adding inorganic fillers into a polymer matrix can elevate ionic conductivity and mechanical strength.^[^ ^18^, ^19^, ^20^ ^]^ Nonetheless, the mismatch of inorganic filler size (typically greater than 50 nm) with the distance between neighboring Li‐ion binding sites in PEO (≈0.5 nm) would only lead to a limited improvement of ionic conductivity in the specific position, causing the inhomogeneous and inferior Li‐ion flux.^[^ ^21^ ^]^ Besides, these CSEs are also unable to escape the trade‐off between mechanical modulus and adhesion.^[^ ^22^ ^]^

Succinonitrile (SN) features a molecular size similar to the unit Li‐ion transfer distance in PEO, combined with the excellent salt‐solvating ability optimizing the chemical environment of Li‐ion, makes SN an ideal filler to facilitate homogeneous and fast Li‐ion flux in PEO‐based CSEs.^[^ ^23^ ^]^ Meanwhile, the addition of SN will not only increase the viscosity of the electrolyte and create adhesive contact between the electrolyte and electrodes, thus reducing the interfacial resistance but also achieve homogeneous and fast Li‐ion flux when the SN content is sufficient.^[^ ^24^, ^25^ ^]^ Notably, the SN content in reported common PEO/SN electrolytes rarely exceeded 10 wt.% to ensure that the CSE could be free‐standing.^[^ ^21^ ^]^ Therefore, the pivotal issue is how to balance the mechanical strength of PEO/SN electrolytes with homogeneous and fast Li‐ion flux.

Recently, laminar membranes constructed by the self‐stacking of 2D nanosheets with high aspect ratios have garnered a lot of attention due to their ultrathin yet robust features.^[^ ^26^, ^27^ ^]^ Especially, laminar membrane, with metal–organic framework (MOF) nanosheets containing coordinatively unsaturated metal sites as building blocks, also exhibits an ability to anchor polar molecules.^[^ ^28^ ^]^ For instance, Fang et al. developed a laminar MOF membrane applied in lithium‐sulfur battery to trap polysulfide through the strong interaction between MOF nanosheets and polysulfide.^[^ ^29^ ^]^ Wang and coworkers utilized the coordination interaction storing SN in a laminar MOF membrane to prepare an ultra‐thin electrolyte with remarkable electrochemical performances.^[^ ^30^ ^]^ These demonstrate the great potential of advanced laminar structure applied to SSEs. Utilizing this superior structure to store viscous‐fluid PEO/SN electrolytes provides a possibility for the realization of SSEs simultaneously possessing the mentioned merits. Besides, the low density of MOF and ultrathin properties are favorable factors for increasing the energy density of ASSLMBs.^[^ ^12^ ^]^

**Scheme 1:**
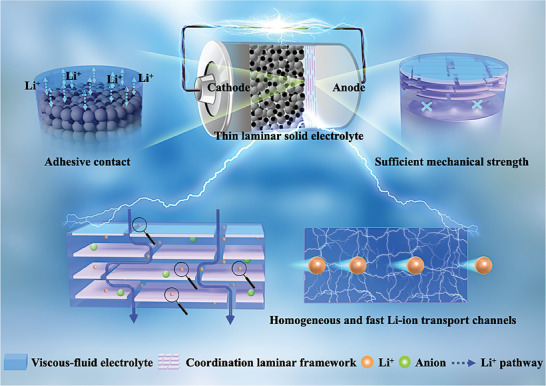
Schematic illustration of thin laminar solid electrolyte with robust and adhesive features as well as homogeneous and fast Li‐ion flux applied in ASSLMBs.

Thereupon, we propose a strategy for designing thin laminar solid electrolytes that can actualize the homogeneous and fast Li‐ion flux while also breaking the trade‐off between mechanical modulus and adhesion by intercalating highly conductive, homogeneous, and viscous‐fluid ionic conductor into robust coordination laminar framework, as seen in **Scheme** **1**. This work gives an example of a 9 µm‐thick laminar solid electrolyte with homogeneous and fast Li‐ion conduction (LSE‐HFC) prepared through filtrating viscous‐fluid PEO/SN/Li salts (molar ratio of SN:EO = 1:4) into robust laminar MOF framework (LMF) formed by self‐stacking of tetrakis(4‐carboxy‐phenyl)porphyrin copper (CuTCPP) nanosheets. We demonstrate that the interaction between coordinatively unsaturated copper sites of CuTCPP and PEO/SN imparts LSE‐HFC mechanical stability. The competing transfer mechanism of [PEO···Li^+^···SN] is verified and works across the entire LSE‐HFC, which facilitates homogeneous Li‐ion flux and low migration energy barriers, endowing LSE‐HFC with high ionic conductivity of 5.62 × 10^−4^ S cm^−1^ and Li‐ion transference number of 0.78 at 25 °C. Meanwhile, LSE‐HFC also obtains an outstanding Young's modulus against punctures and enhanced adhesion force benefiting Li‐ion transfer within the electrolyte/electrodes interfaces, consolidating the uniform Li plating/stripping behavior. Thereupon, the assembled LiFePO_4_ (LFP)/Li cell delivers excellent stability of 600 and 300 cycles at 55 °C, 0.5C and 25 °C, 0.2C, respectively. The LiNi_0.6_Mn_0.2_Co_0.2_O_2_ (NCM622)/Li cell realizes a high energy density of 420.7 Wh kg^−1^ and cycles over 150 cycles with capacity retention of 88.7% at 25 °C and 0.2C.

## 2 Results and Discussion

### 2.1 Preparation and Characterization of LSE‐HFC

**Figure 1:**
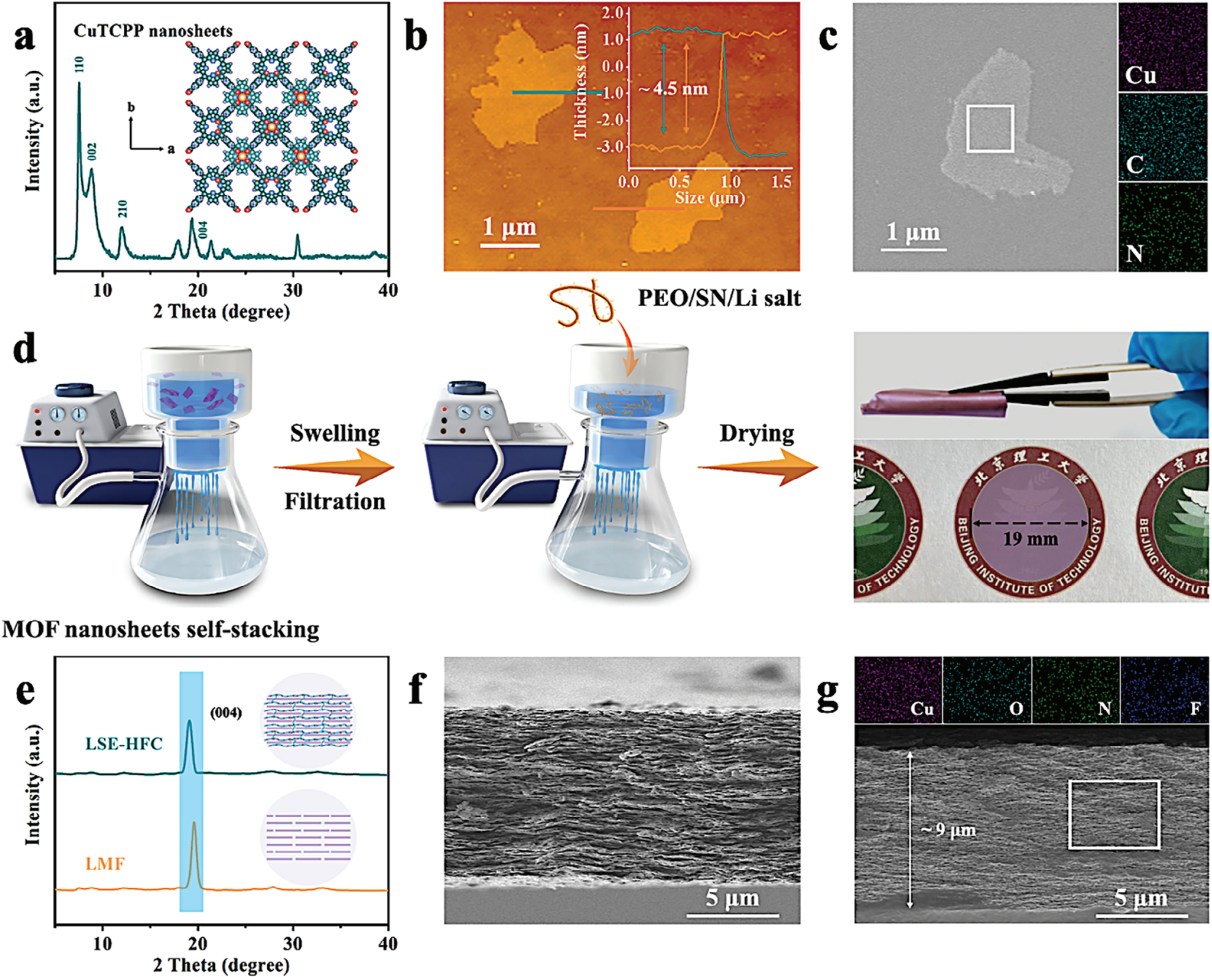
a) XRD pattern, b) AFM image and the corresponding height profiles, and c) SEM image and the corresponding elemental EDS (Cu, C, and N) mappings of CuTCPP nanosheets. d) Schematic illustration of the preparation of LSE‐HFC. e) XRD patterns of LMF and LSE‐HFC. Cross‐sectional SEM images of f) LMF and g) LSE‐HFC (inset displays the corresponding elemental EDS (Cu, O, N, and F) mappings).

The CuTCPP nanosheets were obtained by a surfactant‐assisted synthetic method (Figure S1, Supporting Information).^[^ ^31^ ^]^ FTIR spectra in Figure S2 (Supporting Information) reveal the peaks located at ≈1400 and 1610 cm^−1^, which demonstrates the formation of Cu_2_(COO)_4_.^[^ ^29^ ^]^ The crystal structure of CuTCPP is well confirmed by powder XRD (**Figure** **1**a), and the high‐magnification TEM image in Figure S3 (Supporting Information) displays that the lattice fringes with *d*‐spacings of 0.46 nm match with the (004) plane of CuTCPP. AFM images exhibit that CuTCPP nanosheets possess lateral size of 1−3 µm and thickness of ≈4.5 nm (Figure 1b; Figure S4, Supporting Information), SEM and mapping images demonstrate Cu, C, and N elements are homogeneously dispersed in the nanosheets (Figure 1c). Collectively, these results demonstrate the successful preparation of CuTCPP nanosheets. Noteworthily, the micropores size of CuTCPP nanosheets measured by N_2_ adsorption experiment is ≈0.59 nm (Figure S5, Supporting Information), which is believed to be beneficial for the transport of Li‐ion but limiter for bigger anions owing to the sieving effect, thus contributing to the upgrade of Li‐ion transference number.^[^ ^7^ ^]^

The preparation process of LSE‐HFC is schematically illustrated in Figure 1d. To guarantee that the dispersed solution of CuTCPP nanosheets could readily reassemble into a thin, well‐ordered, and robust film on a nylon substrate, low‐pressure and constant‐rate filtration was first adopted. After undergoing the drying step, LMF was obtained, as shown in Figure S6 (Supporting Information). Here, the well‐ordered laminar structure is crucial to facilitate the intercalation and further continuity of the objects that are subsequently introduced.^[^ ^32^ ^]^ XRD pattern displayed in Figure 1e shows that LMF exhibits almost one peak corresponding to (004) plane, which means that the nanosheets are highly oriented with stacking along the *c*‐axis, forming a regular laminar structure.^[^ ^29^ ^]^ The cross‐sectional SEM image in Figure 1f further confirms the regular orientation of LMF. Following the swelling step, LMF was filled with a low‐concentration PEO‐SN‐LiTFSI‐LiDFOB‐acetonitrile solution (molar ratios of EO:Li and EO:SN of 18:1 and 4:1, respectively) by vacuum filtration method, and then dried for 24 h in the argon‐filled glovebox to obtain LSE‐HFC. 1 wt.% LiDFOB was added to participate in the construction of CEI and SEI.^[^ ^33^ ^]^ The mass ratios of LMF and SN in as‐prepared LSE‐HFC reach 49.7% and 12.3%, respectively (Figure S7, Supporting Information). Noteworthily, the polymer existing in LMF interlayers serves as a deformable buffer, endowing LSE‐HFC with bendable properties (inset in Figure 1d), which will facilitate the assembly of pouch cells.^[^ ^34^ ^]^ The cross‐sectional SEM image in Figure 1g reveals an engorged and compact laminar morphology with a thickness of ≈9 µm, and corresponding elemental EDS (Cu, O, N, and F) mappings demonstrate the uniform distribution of PEO, SN, and Li salts in LSE‐HFC. This thin property is a favorable factor for increasing the energy density of ASSLMBs.^[^ ^12^ ^]^ XRD pattern of LSE‐HFC in Figure 1e also exhibits almost one peak at 19.1°, while the diffraction peaks belonging to PEO, SN, and Li salts are not visibly observed (Figure S8, Supporting Information). This phenomenon should be ascribed to the fact that PEO/SN/Li salts system in LSE‐HFC is nearly amorphous, which will boost Li‐ion conduction. In addition, compared with LMF, the peak corresponding to (004) plane in LSE‐HFC shifts slightly, which might be owing to the occupation of the unsaturated coordination around Cu by PEO/SN.^[^ ^35^ ^]^ The surface morphologies of LMF and LSE‐HFC are observed in Figure S9 (Supporting Information), which reveals a much smoother surface of LSE‐HFC. Meanwhile, the roughness of LSE‐HFC is significantly reduced compared to PEO/LiTFSI and PEO/SN/LiTFSI (Figure S10, Supporting Information), which helps in the close contact of LSE‐HFC with the electrodes.

### 2.2 Exploring the Li‐Ion Chemical Environment in LSE‐HFC

**Figure 2:**
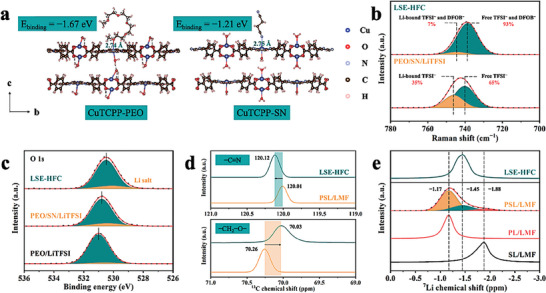
a) DFT calculated adsorption energy of PEO and SN on the CuTCPP nanosheets. b) Raman spectra of PEO/SN/LiTFSI and LSE‐HFC. c) O 1s XPS spectra of PEO/LiTFSI, PEO/SN/LiTFSI, and LSE‐HFC. d) ^13^C NMR spectra of PSL/LMF and LSE‐HFC. e) ^7^Li solid‐state MAS NMR spectra of SL/LMF, PL/LMF, PSL/LMF, and LSE‐HFC.

The ^13^C NMR measurements were carried out to verify the interaction between LMF and PEO/SN (Figure S11, Supporting Information). For PSL/LMF (prepared by filtrating PEO/SN/LiTFSI into LMF), the peaks representing ─CH_2_─O─ in PEO and ─C≡N in SN all shift to lower field compared with PEO/SN/LiTFSI, indicating the reduced electron cloud density around ─CH_2_─O─ and −C≡N and implying that the coordination interaction is more likely to be formed between unsaturated copper sites of LMF and PEO/SN. To further demonstrate this interaction, adsorption energy was calculated using the DFT (**Figure** **2**a). It is revealed that CuTCPP nanosheets, the building blocks of LMF, have negative adsorption energies of −1.67 and −1.21 eV with PEO and SN, respectively, which suggests an excellent adhesion capability between the LMF and PEO/SN. The coordination imparts LMF the ability to store viscous‐fluid PEO/SN electrolyte, which ensures the mechanical stability of LSE‐HFC. Besides, such coordination may reduce the binding effect of ─CH_2_─O─ and −C≡N on Li‐ion, thereby promoting Li‐ion transport.^[^ ^36^ ^]^

Raman spectroscopy was employed to study the dissociation ability of Li salts in SSEs, as shown in Figure 2b. For LSE‐HFC, the peak ≈740 cm^−1^ is assigned to free TFSI^−^ and DFOB^−^ anions, and the peak ≈745 cm^−1^ belongs to coordinated ones. A lower relative content of coordinated anions (7%) can be detected in LSE‐HFC than in PEO/SN/LiTFSI (35%). This result suggests the enhanced dissociation degree of Li salts and optimized Li‐ion chemical environment in LSE‐HFC.^[^ ^7^, ^36^, ^37^ ^]^ Next, the Li‐ion chemical environment in SSEs was explored in detail. As the XPS analysis shown in Figure 2c, the peak of oxygen in the ethylene oxide repeating unit in PEO/LiTFSI is located at 531.0 eV, while those of PEO/SN/LiTFSI and LSE‐HFC shift toward lower binding energy at 530.8 and 530.5 eV, respectively, indicating that the higher electron cloud density around oxygen atoms in PEO is obtained with increasing the SN content.^[^ ^36^ ^]^ A similar phenomenon can be seen in the XPS analysis of N 1s for SSEs (Figure S12, Supporting Information). All the binding energies are calibrated with C 1s at 284.8 eV. These results imply that PEO and SN jointly compete for Li‐ion on the surface of the LSE‐HFC sample. To verify this competing mechanism at an overall level in LSE‐HFC, the ^13^C NMR spectroscopy was conducted. As shown in Figure 2d, compared with PSL/LMF, the peak of ─CH_2_─O─ in PEO shifts to the higher field (70.26–70.03 ppm), and the peak of −C≡N in SN shifts to the lower field (120.01–120.12 ppm) in LSE‐HFC, demonstrating the increased electron cloud density around ─CH_2_─O─ and reduced electron cloud density around ─C≡N.^[^ ^38^ ^]^ This is consistent with the results of the XPS analysis (Figure 2c; Figure S12, Supporting Information), and effectively indicates that the [PEO···Li^+^···SN] system is generated in LSE‐HFC. We further studied the homogeneity of the Li‐ion chemical environment in LSE‐HFC by ^7^Li solid‐state MAS NMR measurements. In this experiment, the effect of the coordination of LMF on the Li‐ion chemical environment was considered. SL/LMF and PL/LMF were prepared by filtrating SN/LiTFSI and PEO/LiTFSI into LMF, respectively. Figure 2e clearly shows three Li‐ion local environments in PSL/LMF: SN phase (−1.88 ppm), PEO phase (−1.17 ppm), and PEO/SN interface (−1.45 ppm).^[^ ^21^, ^25^, ^39^ ^]^ For LSE‐HFC, the peak is located at ≈−1.45 ppm, meaning that Li‐ion in LSE‐HFC is mainly present at the PEO/SN interface. This result suggests that competing mechanism [PEO···Li^+^···SN] works across the entire LSE‐HFC, which facilitates homogeneous Li‐ion flux and low migration energy barriers.

### 2.3 Electrochemical and Mechanical Performances of LSE‐HFC

**Figure 3:**
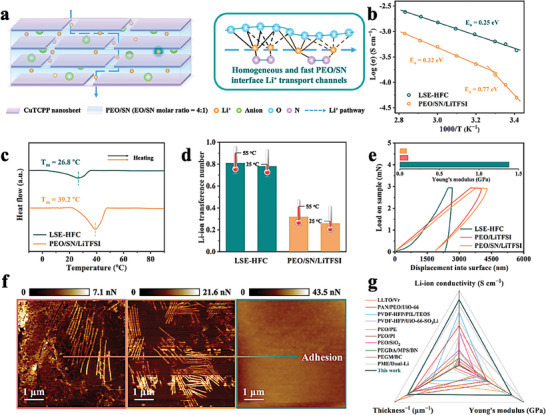
a) Schematic illustration of Li‐ion transfer mechanism in LSE‐HFC. b) The ionic conductivities, c) DSC curves, and d) Li‐ion transference numbers of PEO/SN/LiTFSI and LSE‐HFC. e) load‐displacement curves and (f) adhesion force mapping by AFM of PEO/LiTFSI, PEO/SN/LiTFSI, and LSE‐HFC. (g) Comparison of Li‐ion conductivity, Young's modulus, and thickness with other SSEs in literature, and the data are presented in Table S1 (Supporting Information).

In light of the above results, a schematic illustration of Li‐ion transfer mechanism in LSE‐HFC is shown in **Figure** **3**a. The PEO and SN confined in LMF jointly construct homogeneous and fast Li‐ion transport channels, contributing to the superior Li‐ion transfer ability, which can be assessed in terms of ionic conductivity and Li‐ion transference number ($t_{Li^{+}}$). LSE‐HFC obtains a high ionic conductivity of 5.62 × 10^−4^ S cm^−1^ at 25 °C, 6.3 times higher than that of PEO/SN/LiTFSI (Figure S13, Supporting Information). This apparent gap is attributed to the fact that the improvement of ionic conductivity in PEO/SN/LiTFSI only occurs in specific positions, while short‐range, homogeneous, and fast Li‐ion conduction is implemented across the entire LSE‐HFC, which agrees well with the ^7^Li solid‐state NMR results (Figure 2e). Noteworthily, we investigated the optimal SN/EO molar ratio for ionic conductivity of LSE, and the result is displayed in Figure S14 (Supporting Information). The LSE obtains the highest ionic conductivity when the SN content of SN:EO = 1:4 is added, meaning the complete construction of homogeneous and fast Li‐ion transport channels. Continued addition of SN deteriorates the mechanical stability in addition to not helping to increase the ionic conductivity of the electrolyte.^[^ ^21^, ^25^ ^]^ Arrhenius equation was utilized to calculate the activation energy (*E_a_*) of SSEs (Figure 3b). LSE‐HFC exhibits a lower *E_a_* of 0.25 eV than that of PEO/SN/LiTFSI, implying that transport channels confined in LMF provide a low energy barrier for Li‐ion conduction. As shown in Figure 3d and S15, the $t_{Li^{+}}$ of LSE‐HFC at 55 and 25 °C are stimulated to be ≈0.81 and 0.78, respectively, superior to those of PEO/SN/LiTFSI, which should be ascribed to the size sieving effect of micropores existed in CuTCPP nanosheets that suppress the transportation of large anions. The high $t_{Li^{+}}$ of LSE‐HFC means efficient migration of Li‐ion, which could alleviate the interfacial polarization.^[^ ^7^, ^40^ ^]^ Consequently, due to the high ionic conductivity and $t_{Li^{+}}$, LSE‐HFC exhibits the superior Li‐ion transfer ability. The melting point (*T*_m_) of polymer‐based SSEs is an important reference for setting the operating temperature of assembled ASSLMBs.^[^ ^41^ ^]^ In Figure 3c, the *T*_m_ of LSE‐HFC is as low as 26.8 °C, which means that homogeneous and fast Li‐ion transport channels in amorphous state can be established at RT, further guaranteeing favorable cycling performance of the battery in a wide temperature range. Moreover, LSE‐HFC possesses an excellent electrochemical window of 5.1 V (Figure S16, Supporting Information), indicating the great application potential in high‐voltage batteries.

The mechanical properties of LSE‐HFC were also investigated. Young's modulus of SSEs was measured using nanoindentation, which can mimic the punctuation of lithium dendrite.^[^ ^42^ ^]^ As shown in Figure 3e, LSE‐HFC achieves a high Young's modulus of 1.38 Gpa, which is 13.3 and 15.8 times higher than those of PEO/LiTFSI and PEO/SN/LiTFSI, respectively. The significant improvement should be contributed by the robust LMF formed by self‐stacking of CuTCPP nanosheets with a high aspect ratio, which would help to inhibit the punctuation of lithium dendrite during battery cycling. AFM mapping with a quantitative nanomechanics mode (Figure 3f) was applied to reveal the adhesion force of SSEs.^[^ ^22^ ^]^ The result clearly demonstrates that, in comparison to PEO/LiTFSI and PEO/SN/LiTFSI, the adhesion force of LSE‐HFC is substantially higher, which is explained by both the strong electrostatic attraction and Van der Waals interactions at the interface.^[^ ^43^ ^]^ This enhanced adhesion force actualizes close contact of LSE‐HFC with the electrodes, which will enhance the Li‐ion transfer kinetics within the interfaces and further improve the cycling performance of the battery.^[^ ^7^, ^8^ ^]^ In addition, when compared with other SSEs in literature, LSE‐HFC exhibits better comprehensive properties in terms of Li‐ion conductivity, Young's modulus, and thickness (Figure 3g; Table S1, Supporting Information). To sum up, all these results verify this thin, robust, and adhesive LSE with homogenous and fast Li‐ion conduction.

### 2.4 Cycling Stability of LSE‐HFC

**Figure 4:**
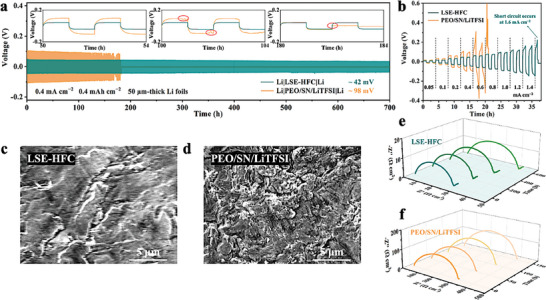
a) Long‐term cycling of Li symmetric cells under 55 °C at 0.4 mA cm^−2^ and 0.4 mAh cm^−2^ (inset shows voltage profiles of cells at 50, 100, and 180 h, respectively). b) Rate performance test of Li symmetrical cells to determine the critical current density. Surface SEM images of Li electrode after lithium plating/stripping in c) LSE‐HFC symmetrical cell and d) PEO/SN/LiTFSI symmetrical cell. The in situ EIS patterns of Li symmetric cells are based on e) LSE‐HFC and f) PEO/SN/LiTFSI.

The as‐prepared SSEs were then tested by Li symmetric cells under 55 °C at 0.4 mA cm^−2^ and 0.4 mAh cm^−2^ to evaluate the stability of Li‐ion plating and stripping on electrodes. The excess Li electrodes would remedy the loss Li and prolong cycling life, leading to a deviation in the actual application.^[^ ^44^ ^]^ Considering that, the thin Li foils (thickness ≈50 µm) were adopted in the symmetric cells (**Figure** **4**a). PEO/SN/LiTFSI exhibits an overpotential of ≈98 mV and relatively stable cycling performance within the first 150 h. After this, a short circuit occurs in PEO/SN/LiTFSI cell with an abrupt drop in voltage at 182 h, which indicates that the irregularly grown lithium dendrite punctures the electrolyte, reflecting both the inhomogeneous and inferior Li‐ion conduction and the unsatisfactory mechanical strength of PEO/SN/LiTFSI. The surface SEM image of the cycled Li electrode in PEO/SN/LiTFSI cell is shown in Figure 4d, as expected, massive irregular lithium dendrite can be observed. On the contrary, the cell assembled with LSE‐HFC shows highly enhanced stability during Li plating and stripping process lasting over 700 h with a low overpotential of ≈42 mV, and its cycled Li electrode exhibits a much flatter morphology (Figure 4c). Furthermore, the cycling performance of the symmetric cells with stepwise increasing current density (from 0.05 to 1.6 mA cm^−2^) is measured to determine the critical current density. As illustrated in Figure 4b, a short circuit occurs in PEO/SN/LiTFSI symmetric cell at a current density of 0.8 mA cm^−2^. For LSE‐HFC cells, the polarization voltage increases almost linearly with current density, while no short‐circuit can be observed up to 1.4 mA cm^−2^, which lays a foundation for pairing with high‐loading cathode.^[^ ^38^, ^42^ ^]^

In addition to the superior Li‐ion conduction and mechanical strength of LSE‐HFC, the improved plating/stripping behavior is also inseparable from the enhanced Li‐ion transfer kinetics within the interfaces arising from the adhesive contact and the formation of a compatible SEI layer. Evidence can be seen in the in‐situ EIS results and XPS analysis. As shown in Figure 4e,f, the pristine charge transfer resistance (*R_ct_*) of Li|LSE‐HFC|Li is just 24.5 Ω cm^2^, which is 11.6 times lower than that of PEO/SN/LiTFSI cell, meaning more efficient Li‐ion transport within the interfaces. Besides, the *R_ct_* of Li|LSE‐HFC|Li is almost unchanged after cycling 200, 400, and 600 h, which could stem from the formation of compatible SEI. To better understand the chemical environment of SEI, the surface of the cycled Li electrode was further characterized by XPS. As shown in Figure S17 (Supporting Information), a considerable amount of Li–F (684.8 eV, F 1s) is detected on Li electrode cycled in Li|LSE‐HFC|Li, which belongs to the decomposition of Li salts.^[^ ^45^ ^]^ B–F (686.1 eV, F 1s; 193.6 eV, B 1s) and B─O (191.3 eV, B 1s) are also observed, which originates from the transformation of LiDFOB.^[^ ^33^ ^]^ The SEI containing these components can realize fast Li‐ion conduction and suppress the growth of lithium dendrite, thus facilitating the cycling stability of LSE‐HFC.^[^ ^36^, ^38^ ^]^ Moreover, at a lower operating temperature of 25 °C, LSE‐HFC cells with limited Li electrodes also cycle stably for more than 400 h at 0.4 and 0.4 mAh cm^−2^ (Figure S18, Supporting Information). These findings highlight the superiority of LSE‐HFC in terms of mechanical properties and Li‐ion transfer ability.

### 2.5 The Performance of LFP ASSLMBs

**Figure 5:**
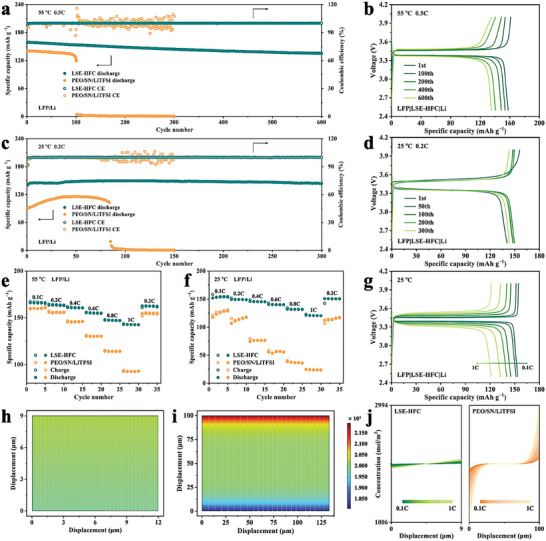
a) Cycling performances of the cells assembled with different electrolytes and b) voltage profiles of LFP|LSE‐HFC|Li at 55 °C and 0.5C. c) Cycling performances of the cells assembled with different electrolytes and d) voltage profiles of LFP|LSE‐HFC|Li at 25 °C and 0.2C. e,f) Rate performances of the cell assembled with different electrolytes at 55 and 25 °C, respectively. g) Voltage profiles of LFP|LSE‐HFC|Li at different rates under 25 °C. h,i) Simulation of Li‐ion distribution at current density of 0.24 mA cm^−2^ in LSE‐HFC and PEO/SN/LiTFSI, respectively. j) Li‐ion concentration along the *y*‐axis in the SSEs at different current densities.

To further explore the potential application of LSE‐HFC, the ASSLMBs with LFP cathode were assembled to first evaluate the cycling performance at 55 °C and 0.5C (1C = 0.68 mA cm^−2^). As shown in **Figure** **5**a, the discharge capacity of PEO/SN/LiTFSI cell decays dramatically after 100 cycles. This unsatisfactory performance should be mainly attributed to the inhomogeneous and inferior Li‐ion conduction as well as the poor mechanical properties of PEO/SN/LiTFSI, which leads to exacerbated battery polarization accompanied by a constant decline in capacity. In comparison, LFP|LSE‐HFC|Li cell shows much better cycling performance, which delivers an initial discharge capacity of 157.8 mAh g^−1^ and undergoes charge/discharge over 600 cycles with capacity retention of 85.7%. The Coulombic efficiency approaches 100% during the long‐term cycling and the energy density reaches 288.4 Wh kg^−1^ (for calculation details, see Table S2, Supporting Information). Corresponding voltage profiles can be seen in Figure 5b and Figure S19 (Supporting Information). The curves of LFP|LSE‐HFC|Li perform much more steadily than those of PEO/SN/LiTFSI cells, showing typical LFP plateaus at ≈3.4 V and maintaining very low overpotentials even at 600 cycles. Such a feature suggests the excellent electrochemical stability of LSE‐HFC cells. Furthermore, the rate performance of these cells was also tested shown in Figure 5e. When cycling at 0.1, 0.2, 0.4, 0.6, 0.8, and 1C, the LFP|LSE‐HFC|Li cell delivers high discharge capacity of 165.7, 163.6, 160.5, 154.8, 147.2, and 142.6 mAh g^−1^, respectively. As the rate comes back to 0.2C, the discharge capacity recovers to 162.4 mAh g^−1^, demonstrating excellent reversibility. Voltage profiles at different rates in Figure S20 (Supporting Information) also confirm the superior rate performance of LFP|LSE‐HFC|Li cell. These results reveal that the homogeneous and fast Li‐ion conduction combined with the favorable mechanical properties endow LSE‐HFC with the superior performance of LFP ASSLMBs.

The properties discussed above show obvious advantages of the LSE‐HFC for application in ASSLMBs at RT. Hence, the cycling performance of LFP/Li cells at 25 °C was evaluated under 0.2C (1C = 1.20 mA cm^−2^). Figure 5c reveals that the discharge capacity of PEO/SN/LiTFSI cell drops rapidly and closes to zero capacity after 90 cycles, while LFP|LSE‐HFC|Li cell achieves an average reversible capacity of 147.3 mAh g^−1^ as well as high average Coulombic efficiency of 99.9% over the first 300 cycles and exhibits an energy density of 343.8 Wh kg^−1^ (Table S2, Supporting Information). This cycling performance is comparable with reported SSEs under similar current density.^[^ ^7^, ^36^, ^38^ ^]^ The decreased potential polarization (from 187 mV in the 1st cycle to 168 mV in the 300th cycle) in the charge–discharge curves, as shown in Figure 5d, indicates the excellent interfacial stability of the LSE‐HFC toward both LFP cathode and Li anode. We further cycled these LFP/Li cells at various current densities from 0.1 to 1C (Figure 5f). Under the current density of 1C, the discharge capacity of LSE‐HFC cell can reach 121.2 mAh g^−1^, much higher than that of PEO/SN/LiTFSI cell (24.8 mAh g^−1^). Corresponding voltage profiles at different rates shown in Figure 5g and Figure S21 (Supporting Information) also verify the excellent rate performance of LFP|LSE‐HFC|Li cell at 25 °C and 0.2C. Based on the above results, it can be concluded that: 1) The adhesive contact between the LSE‐HFC and the electrodes improves the Li‐ion transfer kinetics within the interfaces. 2) The sufficient mechanical strength of LSE‐HFC effectively inhibits the growth of lithium dendrite. 3) The superior Li‐ion transfer ability of LSE‐HFC leads to homogeneous and fast Li‐ion flux. These merits permit the excellent electrochemical performance of LFP ASSLMBs assembled with LSE‐HFC. To visualize this Li‐ion flux in both LFP/Li cells, the distribution of Li‐ion at different current densities was simulated by COMSOL Multiphysics (Figure 5h−j; Figure S22, Supporting Information).^[^ ^42^ ^]^ All the simulation parameters were based on the practical electrochemical parameters of the LFP/Li coin cell at 25 °C. Obviously, the inhomogeneous spatial distribution of Li‐ion concentration intensifies with the increase of current density, and LSE‐HFC features a more homogeneous Li‐ion gradient distribution than PEO/SN/LiTFSI, exhibiting the homogeneous and fast Li‐ion flux in LSE‐HFC cell.

### 2.6 The Performance of NCM ASSLMBs

**Figure 6:**
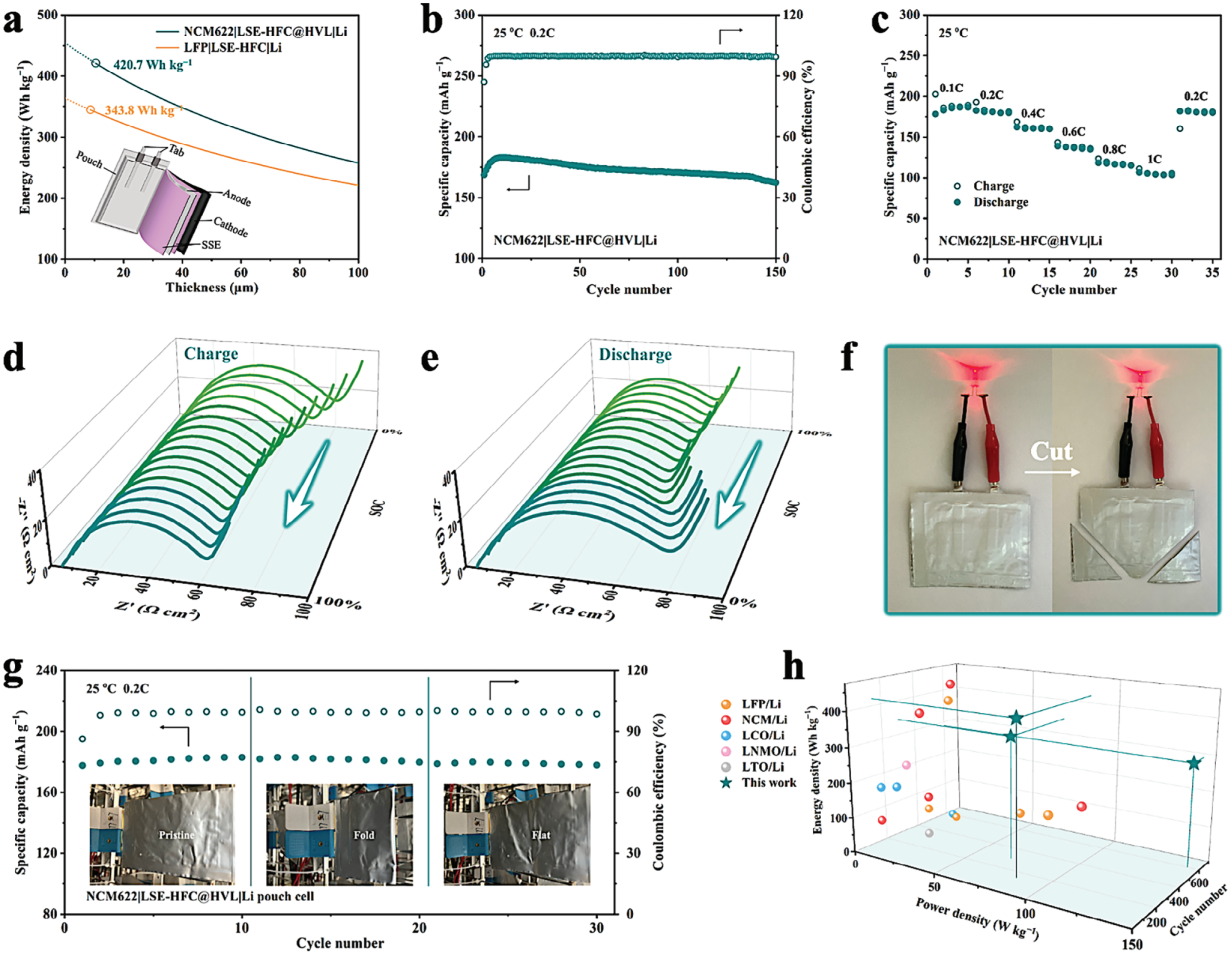
a) Gravimetric energy density as a function of the thickness of LSE‐HFC and LSE‐HFC@HVL employing LFP cathode and NCM622 cathode, respectively (inset displays an idea pouch cell model). b) Cycling performance at 25 °C and 0.2C and c) rate performance at 25 °C of NCM622|LSE‐HFC@HVL|Li cell. In situ EIS during d) charging process and e) discharging process of NCM622|LSE‐HFC@HVL|Li cell at 2.8−4.3 V. f) A structural NCM622|LSE‐HFC@HVL|Li pouch cell lighting a LED bulb after cutting test. g) Cycling performance of the assembled pouch cell at 25 °C and 0.2C. h) Comparison of energy density, power density, and cycling life with other batteries in literature, and the data are presented in Table S3 (Supporting Information).

In pursuit of the high energy density of ASSLMBs, we further matched SSE to a limited Li anode and a high‐loading NCM cathode. Although the electrochemical window of LSE‐HFC satisfies the voltage adaptation requirement, the highly oxidized Ni^4+^ (Co^4+^ or Mn^4+^) with catalytic effect on the surface of NCM cathode active particles induces the decomposition of PEO electrolyte.^[^ ^46^ ^]^ This not only causes the local path failure of Li‐ion transport in electrolytes but also disrupts the crystal structure of cathode active materials.^[^ ^47^ ^]^ To cope with this hindrance for application, the polyacrylonitrile (PAN)‐based electrolyte with the low highest occupied molecular orbital (HOMO) energy level was selected as a high‐voltage layer (HVL) to coat on the surface of the LSE‐HFC against the cathode side, named as LSE‐HFC@HVL. This strategy has been proven to be effective in improving the electrochemical stability of PEO electrolytes in NCM cells.^[^ ^42^ ^]^ A cross‐sectional SEM image of LSE‐HFC@HVL is shown in Figure S23 (Supporting Information), the thickness of HVL is ≈1.5 µm. As expected, such a thin and lightweight electrolyte paired with a limited Li anode and a high‐loading NCM cathode exhibits a high energy density of 420.7 Wh kg^−1^ (**Figure** **6**a; Table S2, Supporting Information). Besides, LSE‐HFC@HVL also obtains a high ionic conductivity of 5.28 × 10^−4^ S cm^−1^ at 25 °C (Figure S24, Supporting Information). The cycling performance of NCM622|LSE‐HFC@HVL|Li cell was evaluated at 25 °C and 0.2C (1C = 1.30 mA cm^−2^). Figure 6b reveals that a discharge capacity of 182.8 mAh g^−1^ is obtained after activation and a capacity retention of 88.7% is retained after 150 cycles. The smooth charge/discharge profiles show that there is no side reaction when charged up to 4.3 V (Figure S25, Supporting Information).^[^ ^38^ ^]^ Furthermore, NCM622|LSE‐HFC@HVL|Li cell also shows a decent high‐rate performance in Figure 6c. The discharge capacities are 186.7, 182.5, 162.2, 138.8, 117.8, and 106.7 mAh g^−1^ at 0.1, 0.2, 0.4, 0.6, 0.8, and 1C, respectively. When the rate restores to 0.2C, the specific capacity quickly returns to 181.6 mAh g^−1^. The cycling performance at 55 °C and 0.5C was also evaluated. NCM622|LSE‐HFC@HVL|Li cell delivers a discharge capacity of 178.6 mAh g^−1^ after 200 cycles with a capacity retention of 96.4% in 2.8–4.3 V (Figure S26, Supporting Information). These results suggest that homogeneous and fast Li‐ion flux combined with sufficient mechanical strength and enhanced adhesion force of LSE‐HFC@HVL contribute to the excellent performances of NCM ASSLMBs. Besides, the interfacial compatibility between the NCM cathode and the electrolyte also plays an important role in acquiring such excellent performances. According to the in situ EIS results in Figure 6d,e, the semicircles in the Nyquist plots are related to the *R_ct_* from the electrodes/electrolyte interfaces.^[^ ^48^ ^]^ The *R_ct_* undergoes slight variations, first decreasing and then rising with the increase of state of charge (SOC). The observed alteration should be attributed to the change in ionic and electronic conductivity of the cathode in the process of delithiation. During the discharge process, the *R_ct_* almost displays reversible variations. The higher impedance for the discharged cell with 0% SOC compared with the fresh cell should be attributed to the diffusion limitation in the NCM particles after discharge. It can be concluded that the *R_ct_* of NCM622|LSE‐HFC@HVL|Li cell is relatively stable at different SOC, and the total area‐specific resistance is relatively low (<80 Ω cm^−2^) during the charge/discharge process. Nyquist plots for NCM622 cells cycled at 25 °C and 0.2C were collected at the 0% SOC (Figure S27, Supporting Information). It can be observed that the *R_ct_* exhibits a negligible resistance increase within ten cycles. These results indicate the efficient Li‐ion conduction at electrodes/electrolyte interfaces and the formation of compatible CEI. Subsequently, the surface chemistry and morphology of NCM622 particles after ten cycles are observed by XPS and TEM, respectively, as shown in Figure S28 (Supporting Information). The Li_x_BO_y_F_z_ arising from the decomposition of LiDFOB in LSE‐HFC@HVL occurs at 191.3 eV, participating in the formation of compatible CEI, which is a homogeneous and thin protection layer with an average thickness of ≈10 nm in favor of improving the interfacial stability.^[^ ^33^ ^]^ At the anode side, the surface morphology of cycled Li foil is smooth and flat, which again reflects the interfacial stability between the electrolyte and electrodes (Figure S29, Supporting Information). Compared with the battery performances in literature, the LFP/Li cell and NCM622/Li cell assembled with LSE‐HFC and LSE‐HFC@HVL, respectively, all exhibit superior comprehensive performances in terms of energy density, power density, and cycling life (Figure 6h; Table S3, Supporting Information) Additionally, the scale‐up potential of LSE‐HFC@HVL was assessed via a pouch cell constructed by Li anode and NCM622 cathode. The pouch cell exhibits excellent safety performance and has the ability to light up a red LED bulb when suffering from cutting (Figure 6f). Figure 6g reveals that the pouch cell delivers an initial discharge capacity of 179.2 mAh g^−1^ and can run normally over 30 cycles without obvious capacity decay when being folded.

## 3 Conclusion

In this study, the fabrication of 9 µm‐thick laminar solid electrolyte featuring homogeneous and fast Li‐ion flux is reported. We confirm that the interaction between coordinatively unsaturated copper sites of CuTCPP and PEO/SN imparts LSE‐HFC mechanical stability. The robust LMF, formed by the self‐stacking of CuTCPP nanosheets with a high aspect ratio, allows LSE‐HFC to achieve a high Young's modulus of 1.38 Gpa. Meanwhile, viscous‐fluid PEO/SN electrolyte confined in LMF provides homogeneous and fast Li‐ion transport channels and adhesive contact with electrodes. Specifically, the competing transfer mechanism of [PEO···Li^+^···SN] is verified and works across the entire LSE‐HFC to form such Li‐ion transport channels, endowing LSE‐HFC with high ionic conductivity of 5.62 × 10^−4^ S cm^−1^ and Li‐ion transference number of 0.78 at 25 °C. The adhesive contact between LSE‐HFC and electrodes facilitates efficient Li‐ion conduction at interfaces, consolidating the uniform Li plating/stripping behavior. Thereupon, the assembled LFP/Li cell delivers excellent stability of 600 and 300 cycles at 55 °C, 0.5C, and 25 °C, 0.2C, respectively. The NCM622/Li cell realizes a high energy density of 420.7 Wh kg^−1^ and cycles over 150 cycles with capacity retention of 88.7% at 25 °C and 0.2C. Confining highly conductive, homogeneous, and viscous‐fluid electrolytes into a robust coordination laminar framework can be considered a universal strategy for fabricating thin SSEs with homogeneous and fast Li‐ion flux while breaking the trade‐off between mechanical modulus and adhesion.

## 4 Experimental Section

### Materials and Chemicals

PEO (Mw = 600 000), PAN (Mw = 150 000), SN (99.0%), lithium bis(trifluoromethanesulfonyl)imide (LiTFSI, 99.9%), lithium difluoro(oxalato)borate (LiDFOB, 99.0%), acetonitrile (99.0%), and N, N‐dimethylformamide (DMF, 99.9%) were purchased from Macklin. Copper nitrate trihydrate (Cu(NO_3_)_2_·3H_2_O, 99.0%), polyvinylpyrrolidone (PVP, Mw = 40 000), trifluoroacetic acid (TFA, 99.5%), tetrakis(4‐carboxyphenyl)porphyrin (TCPP, 97.0%), and ethanol (99.7%) were supplied by Sigma‐Aldrich. LFP, NCM622, super‐P, and aluminum (Al) foil were obtained from Canrd New Energy Technology Co., Ltd. Lithium foil was supplied by China Energy Lithium Co., Ltd. Polyvinylidene fluoride (PVDF, Mw = 1500 000) and N‐methyl‐2‐pyrrolidone (NMP, 99.5%) were purchased from Aladdin Chemical Co., Ltd. Deionized water was utilized throughout the experiment.

### Synthesis of CuTCPP Nanosheets

CuTCPP nanosheets were synthesized according to a previous report.^[^ ^31^ ^]^ Cu(NO_3_)_2_·3H_2_O (30.2 mg) and PVP (80.0 mg) were dissolved in the mixture of DMF (45 mL) and ethanol (15 mL) followed by adding TFA (0.05 mL) under stirring for 15 min. Then, TCPP (33.0 mg) dissolved in the mixture of DMF (45 mL) and ethanol (15 mL) was added under stirring for 20 min. After that, the mixed solution was heated to 80 °C, and the reaction was continued for 3 h. The resultant solution was centrifuged at 8000 rpm for 10 min, followed by washing with ethanol (three times) and deionized water (one time). Through freeze drying for 12 h, the CuTCPP nanosheets were obtained.

### Preparation of LSE‐HFC, LSE‐HFC@HVL PEO/SN/LiTFSI CSE, PEO/LiTFSI SPE, PSL/LMF, PL/LMF, and SL/LMF

The CuTCPP nanosheets were dispersed in ethanol and subsequently sonicated for 30 min. After the dispersion was rested adequately, filtrating the supernatant on a nylon substrate to form well‐ordered LMF. Subsequently, LMF was swelled in acetonitrile solution for 1 h, which ensured adequate swelling for a larger interlayer distance. The PEO, SN, LiTFSI, LiDFOB were dissolved in acetonitrile (molar ratios of EO:Li and EO:SN were 18:1 and 4:1, respectively), and the content of LiDFOB added was ≈1 wt.%. The resultant solution was filtrated into LMF, followed by drying for 24 h at 55 °C in an argon‐filled glovebox to obtain LSE‐HFC.

The PAN, SN, and LiDFOB (5:2:1, optimal mass ratio) were dissolved in DMF and stirred at 60 °C to obtain a homogeneous slurry. Then casting the slurry on the surface of LSE‐HFC as HVL, followed by drying for the first 24 h at 40 °C and for the next 24 h at 80 °C in the argon‐filled glovebox. The as‐prepared electrolytes, denoted as LSE‐HFC@HVL, were applied in NCM622/Li cells testing.

The PEO/LiTFSI SPE and PEO/SN/LiTFSI CSE were prepared by a solution casting method. The PEO and LiTFSI (EO/Li molar ratio of 18:1) were dissolved in acetonitrile under stirring for 6 h. The resultant mixed solution was cast onto a polytetrafluoroethylene (PTFE) plate and then dried at 40 °C for 12 h under argon atmosphere and kept at 55 °C for 12 h under vacuum to obtain PEO/LiTFSI SPE. The preparation process of PEO/SN/LiTFSI CSE was similar to that of the SPE, and the SN content was ≈10 wt.%. All procedures were carried out in an argon‐filled glovebox.

The preparation process of PSL/LMF, PL/LMF, and SL/LMF was to filtrate PEO/SN/LiTFSI, PEO/LiTFSI, and SN/LiTFSI solutions into LMF, respectively, followed by drying for 24 h at 55 °C in the argon‐filled glovebox. These three electrolytes were utilized for nuclear magnetic resonance (NMR) measurement.

### Characterization

Fourier transform infrared (FTIR, Nicolet MAGNA‐IR560) spectra were used to analyze the chemical information of CuTCPP nanosheets at wave numbers of 400–4000 cm^−1^. The Brunauer–Emmett–Teller (BET) surface area and pore size distribution of CuTCPP nanosheets were calculated from the N_2_ adsorption isotherms. High‐resolution transmission electron microscopy (TEM, FEI Talos F200S) operating at 200.0 kV was applied to observe the lattice fringes of CuTCPP nanosheets and the morphology of cycled NCM622 particles. Atomic force microscopy (AFM, Bruker Dimension FastScan) was used to test the thickness and lateral size distribution of CuTCPP nanosheets, the surface roughness, and the adhesive force of SSEs. X‐ray diffraction (XRD, Bruker D8 Advance ECO) model was used to analyze the crystallization behavior of PEO, SN, LiDFOB, LiTFSI, CuTCPP nanosheets, LMF, and LSE‐HFC. The microstructure and morphology of CuTCPP nanosheets, LMF, LSE‐HFC, HVL, and cycled lithium anode were detected by scanning electron microscopy (SEM, JSM‐7500F) operating at 5.0 kV after the samples (except for lithium anode) were sputtered with gold, and the corresponding elemental analysis was performed by energy dispersive spectroscopy (EDS, X‐max, Oxford Instruments) operating at 20.0 kV after the samples were sputtered with gold. The thermogravimetric analysis (TGA) of electrolytes was taken using TGA‐50 SHIMADZU from RT to 800 °C with a heating rate of 10 °C min^−1^ under a nitrogen atmosphere. The ^7^Li solid‐state magic angle spinning (MAS) NMR spectra were recorded on Bruker AVANCE III‐500 with a 2.5 mm Bruker HXY probe at Larmor frequencies of 194.4 MHz. ^7^Li chemical shift was calibrated relative to LiCl at 0 ppm. ^13^C NMR spectra were performed using Bruker AVANCE III‐600 of 150.9 MHz, and CDCl_3_ was selected as solvent at RT. X‐ray photoelectron spectroscopy (XPS, Thermo Scientific ESCA Lab 250Xi) was carried out to detect the chemical components and status of SSEs, solid electrolyte interphase (SEI), and cathode electrolyte interphase (CEI). The chemical environments of SSEs were checked by the Raman spectrometer (HR800 Raman spectrometer, Horiba Jobin‐Yvon Ltd) with an exciting laser of 532 nm. The crystallinity and thermal properties of SSEs were detected through differential scanning calorimetry (DSC, STA 449 F3 Jupiter) with a heating rate of 10 °C min^−1^ under a nitrogen atmosphere. Agilent Nano Indenter G200 was used to measure the Young's modulus during nanoindentation experiments. A 200 nm‐diameter rigid cylinder indenter and 3 mN maximum pressure were applied. The Young's modulus (*E*) can be calculated by the following two equations:

(1) $$E_{r}\;=\;\frac{S\sqrt{\pi }}{2\sqrt{A}}$$

(2) $$\frac{1}{E_{r}}\;=\;\frac{1-\;v^{2}}{E}\;+\;\frac{1-\;{v_{i}}^{2}}{E_{i}}$$

where *E*_r_ is the reduced modulus (GPa), *S* is the initial slope of the unloading curve, *A* is the projected area of the elastic contact at peak displacement (nm^2^), *E*_i_ and *v*_i_ are Young's modulus (GPa), and Poisson's ration of the probe, respectively, and *v* is the Poisson's ration of the tested samples.^[^ ^49^ ^]^

### Electrochemical Analysis

The resistances of SSEs were measured on an electrochemical workstation (CHI660E, Shanghai). Assembling stainless steel (SS)|SSEs|SS cells and then testing by electrochemical impedance spectrum (EIS) with frequency ranging from 105 to 0.1 Hz and temperature ranging from 20 to 80 °C. The ionic conductivities (σ) of SSEs were calculated by the following equation:

(3) $$\sigma \;=\;\frac{L}{RA}$$

where *L*, *R*, and *A* represent the thickness (cm), resistance (Ω), and surface area (cm^2^) of the SSEs, respectively. Activation energy *E_a_* for ion transfer in SSEs can be calculated according to the Arrhenius equation:

(4) $$\sigma \;=\;A\;exp\left(\frac{-\;E_{a}}{RT}\right)$$

where *A* is pre‐exponential factor, *R* is the gas constant (J mol^−1^ K^−1^), *T* is the temperature (K). To test the electrochemical stability of the SSEs, the linear sweep voltammetry (LSV) was investigated employing SS|SSEs|Li at a scan rate of 1 mV s^−1^ from open circuit voltage to 7 V. The Li‐ion transference number ($t_{Li^{+}}$) was determined by the electrochemical combination method of direct current (DC) polarization/alternating current (AC) impedance employing a symmetric cell of Li|SSEs|Li. The $t_{Li^{+}}$ was calculated by the following equation:

(5) $$t_{Li^{+}}\;=\;\frac{I_{s}\;\left(\Delta V-\;I_{0}R_{0}\right)}{I_{0}\;\left(\Delta V-\;I_{s}R_{s}\right)}$$

where the value of applied voltage (*ΔV*) is 10 mV, *I_o_* and *I*_s_ are the initial current and steady current during the DC polarization process, respectively, and *R_o_* and *R*_s_ are the resistance values of electrolytes before and after DC polarization, respectively. Symmetric Li|SSEs|Li cells were applied to test the stability of Li plating/stripping and critical current density (CCD). The in situ EIS test was carried out on the Li symmetric cells under 0.4 mA cm^−2^, 0.4 mAh cm^−2^ at 55 °C, and the data were selected every 25 cycles. All batteries were assembled in the argon‐filled glovebox.

### Batteries assembly and Measurement

The cathode slurry was obtained by adequately stirring LFP/NCM622 powder, super‐P, and PVDF with a mass ratio of 8:1:1 in NMP solvent. The obtained slurry was coated on Al foil followed by vacuum drying at 80 °C for 24 h. The prepared electrodes were compacted by a roller press to obtain LFP or NCM622 cathodes with a loading of 4 mg cm^−2^ applied in cells operated at 55 °C and 7 mg cm^−2^ applied in cells operated at 25 °C. The batteries were assembled into CR2032 coin cells. The cycling performances of LFP cells at 0.5 and 0.2C were obtained at 55 and 25 °C with a voltage ranging from 2.5 to 4.0 V, respectively, and the cycling performance of NCM622 cells was tested under 0.2C at 25 °C with a voltage ranging from 2.8 to 4.3 V. The rate performances of such cells were acquired from 0.1 to 1.0C at 55 and 25 °C, respectively. The in‐situ EIS test was performed on the NCM622 cell, and the data were recorded every 0.1 V. The performance of NCM622 pouch cells with active material loading of 7 mg cm^−2^ was also tested.

### Computational Methods

The calculations of binding energy between CuTCPP nanosheets and PEO/SN were conducted using density functional theory (DFT) by the projector‐augmented wave method (PAW) in the Vienna ab initio Simulation Package (VASP). For the electron‐to‐electron interaction, the exchange‐correlation function of the generalized gradient approximation (GGA) with Perdew–Burke–Ernzerh (PBE) parametrization was employed. The binding energy (*E_b_*) was calculated by the following equation:

(6) $$E_{b}\;=\;E_{s,\;a}\;-\;E_{s}\;-\;E_{a}$$

where *E*_s,a_ is the total energy of CuTCPP nanosheets with absorbed PEO or SN, *E*_s_ is the energy of free CuTCPP nanosheets, *E_a_* is the energy of free PEO or SN.

Implementing the physical models of Electrostatic and Transport of Diluted Species in COMSOL Multiphysics, the simulation of modeling of current distribution and Li‐ion redistribution was carried out. The LSE‐HFC and PEO/SN/LiTFSI CSE were modelled in 2D planes with dimensions of 12 µm × 9 µm and 135 µm × 100 µm, respectively. The lower and upper boundaries were the interfaces between the SSEs and electrodes, where the cathode and anode were hidden though. The lithium ions diffusion coefficient was calculated by the Nernst–Einstein equation. The initial Li concentration of 2 mol L^−1^ in the SSE was used. Current densities of 0.1–1C (1C = 1.2 mA cm^−2^) were applied and the Li‐ion gradient in the electrolyte reached its steady state at 60 s already.

## Conflict of Interest

The authors declare no conflict of interest.

## Supporting information

Supporting Information

## Data Availability

Research data are not shared.
