# Supplementary material for: Robust and Adhesive Laminar Solid Electrolyte with Homogenous and Fast Li‐Ion Conduction for High‐Performance All‐Solid‐State Lithium Metal Battery
Source: Adv Sci (Weinh). 2024 Jun 14;11(30):2404307. doi: 10.1002/advs.202404307 (PMC11321704; doi:10.1002/advs.202404307)
Supplement: Supplementary file 1 — Supporting Information [file ADVS-11-2404307-s001.docx]

Supporting Information

**Robust and adhesive laminar solid electrolyte with homogenous and fast Li-ion conduction for high-performance all-solid-state lithium metal battery**

*Shiyuan Guo, Yuefeng Su***, Kang Yan, Chenying Zhao, Yun Lu, Haoyu Wang, Jinyang Dong, Ning Li, Yun Liu, Yibiao Guan, Feng Wu, Lai Chen**


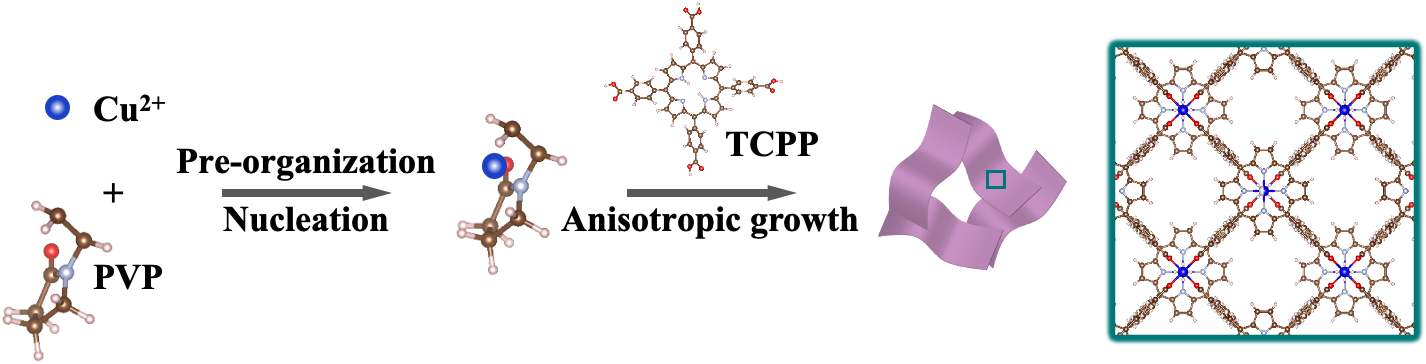


**Figure S1.** Schematic of synthesis process of CuTCPP nanosheets.


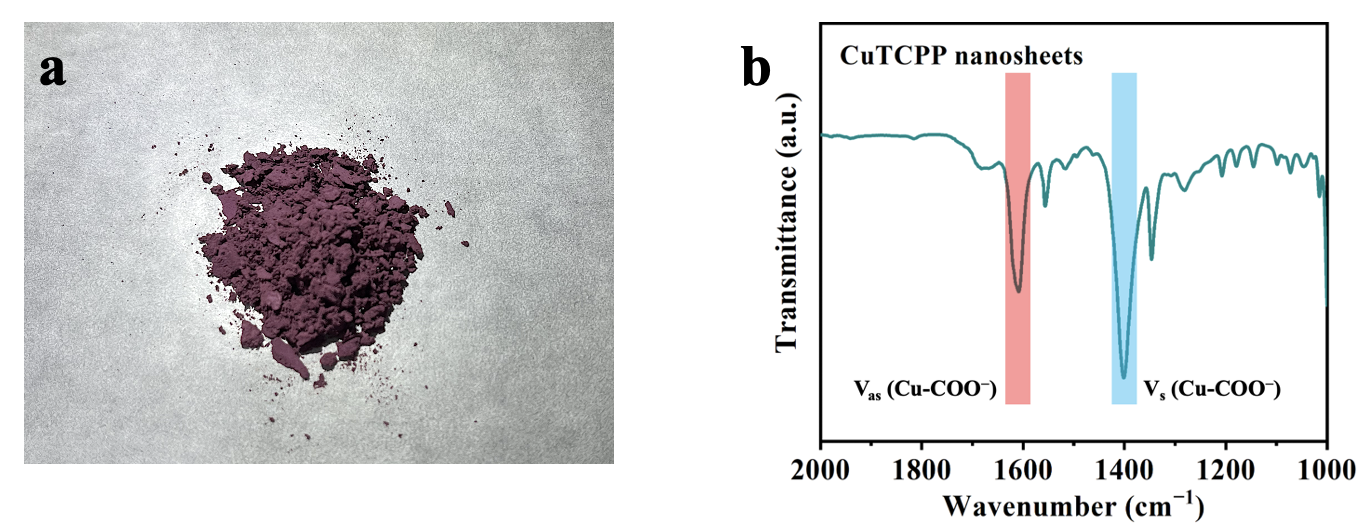


**Figure S2.** (a) Digital photo and (b) FTIR spectra of CuTCPP nanosheets.


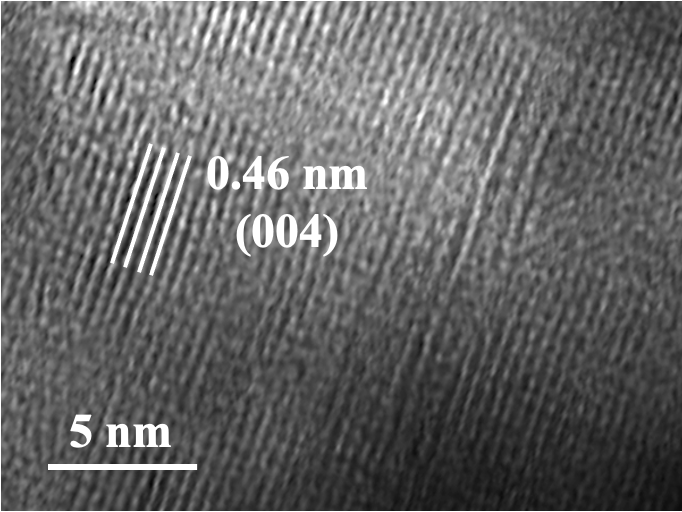


**Figure S3.** High-resolution TEM image of CuTCPP nanosheets.


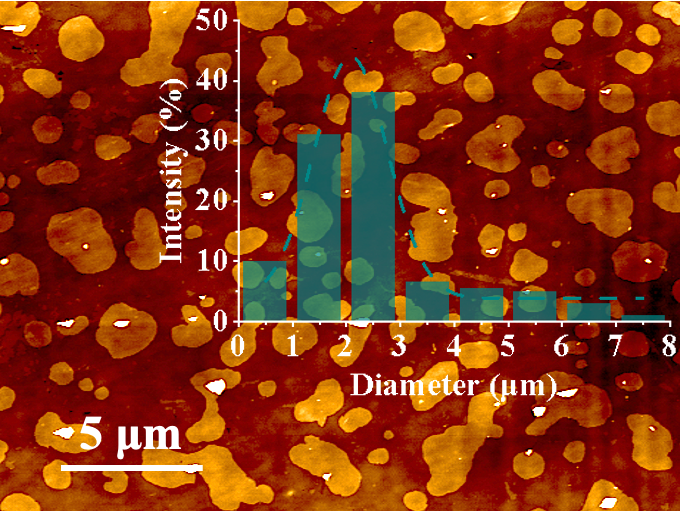


**Figure S4.** The morphology of CuTCPP nanosheets (inset: lateral size distribution of nanosheets).


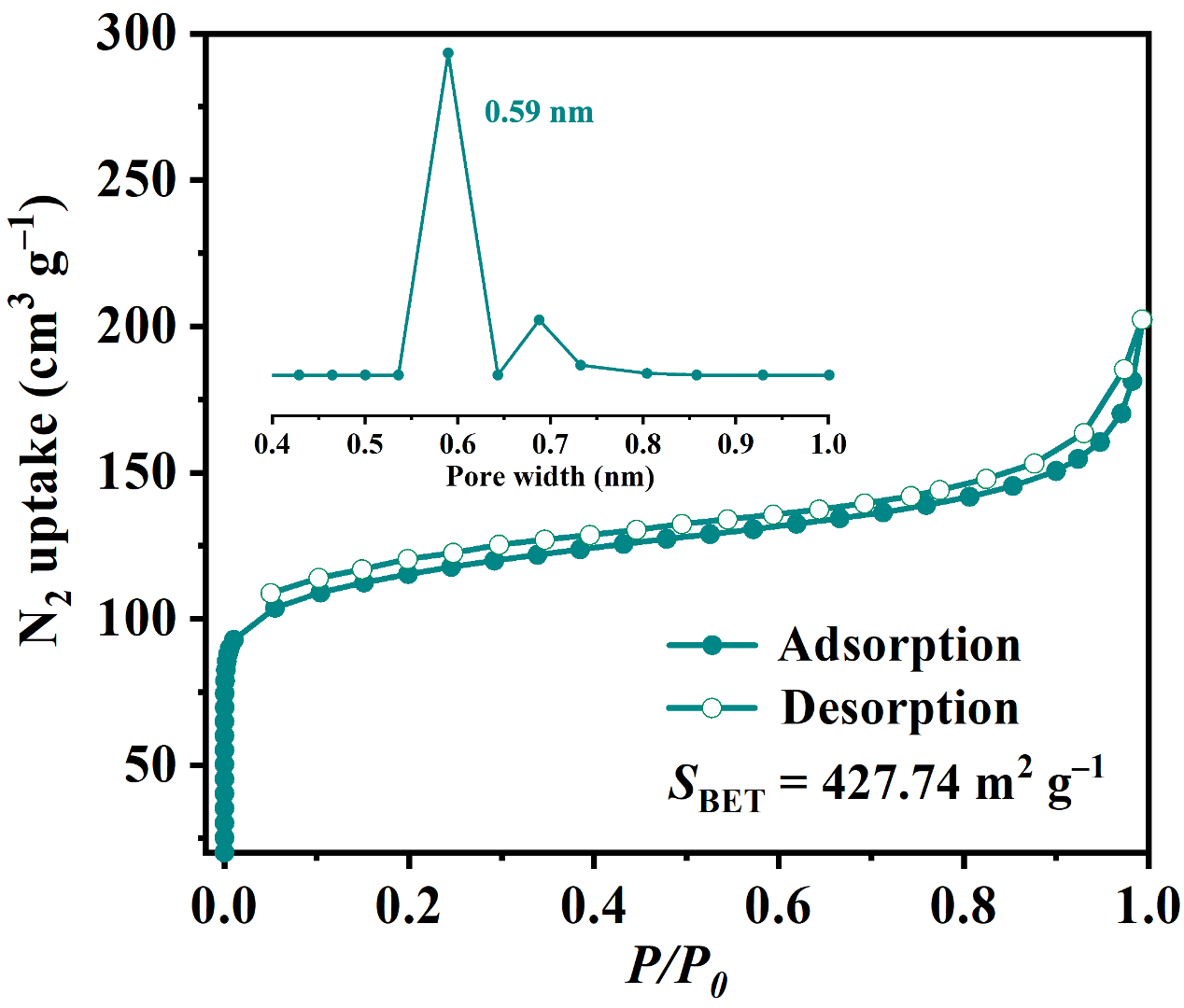


**Figure S5.** The N_2_ adsorption isotherms of CuTCPP nanosheets (inset: pore size distribution).


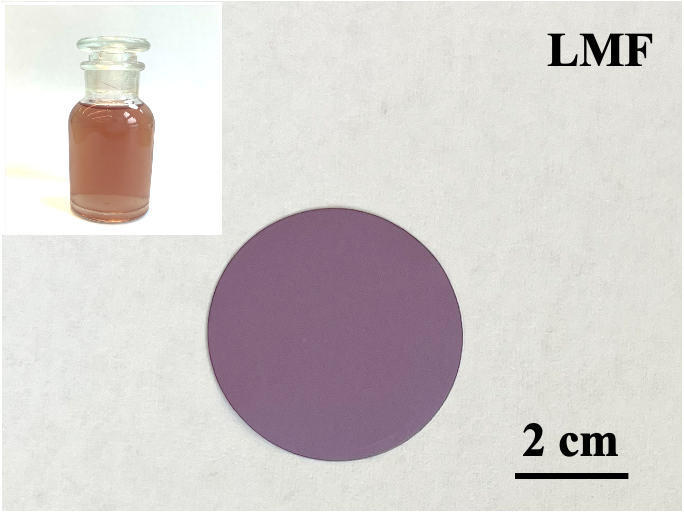


**Figure S6.** The digital photo of LMF (inset: the dispersed solution of CuTCPP nanosheets).


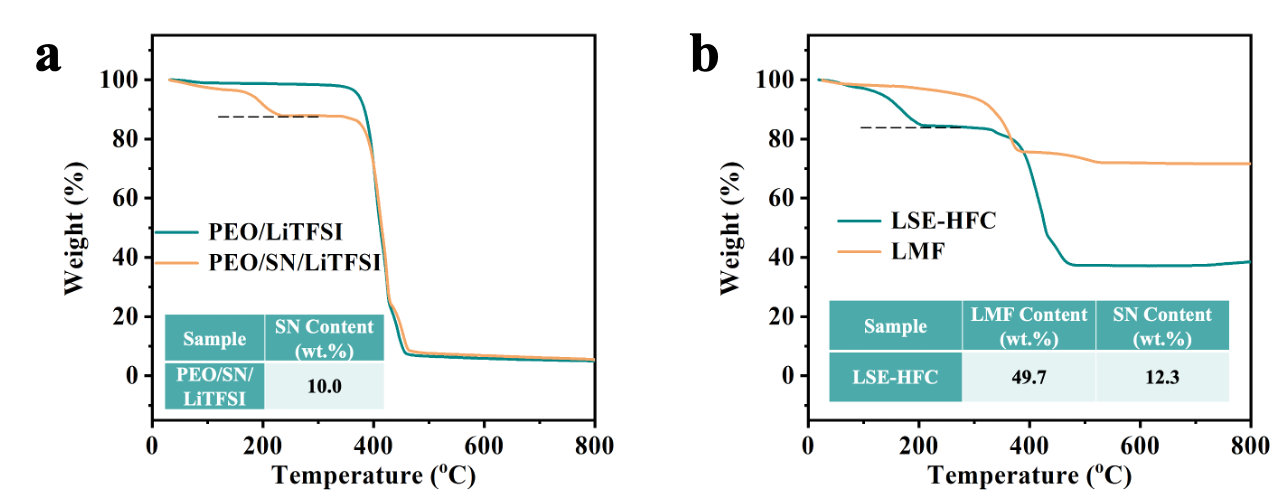


**Figure S7.** Thermogravimetric analysis of (a) PEO/LiTFSI and PEO/SN/LiTFSI and (b) LSE-HFC and LMF.


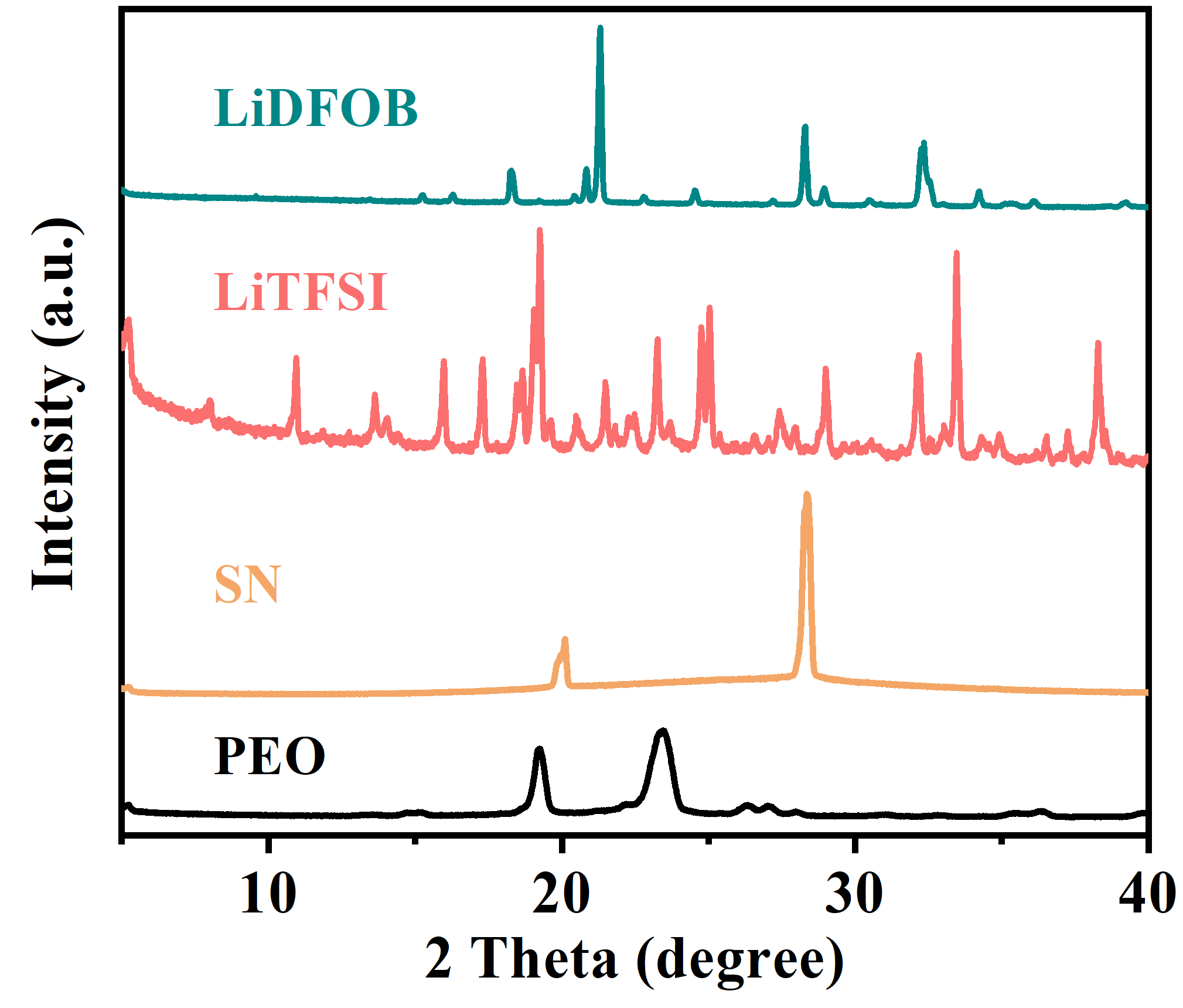


**Figure S8.** XRD patterns of PEO, SN, LiTFSI, and LiDFOB.


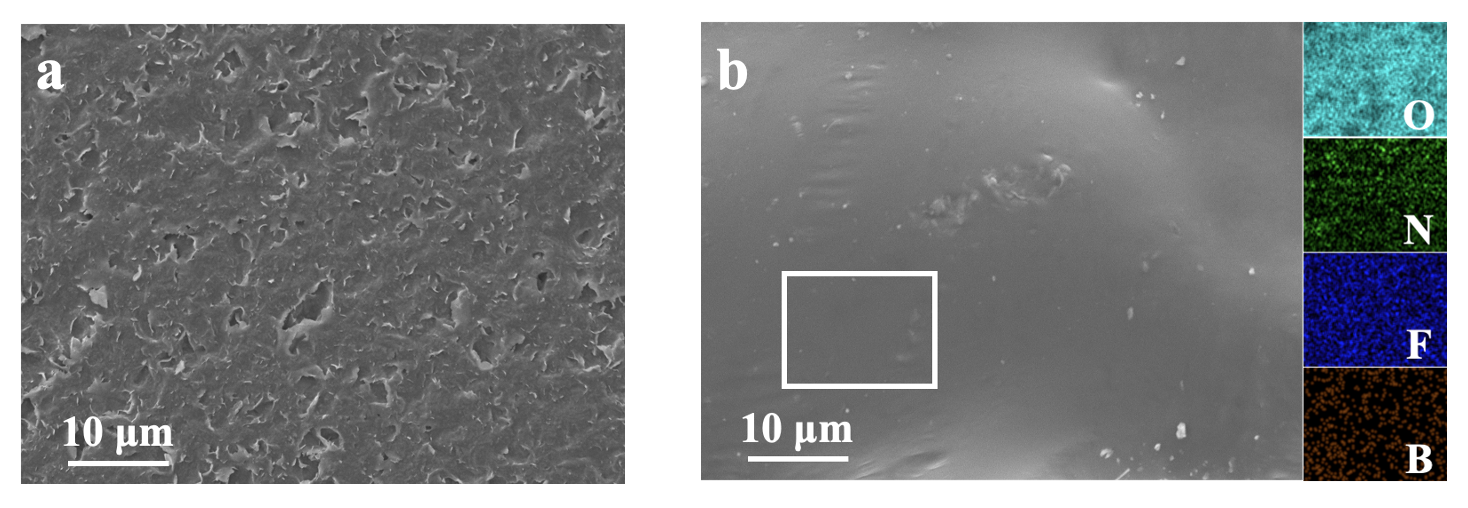


**Figure S9.** Surface SEM images of (a) LMF and (b) LSE-HFC (inset: the corresponding elemental EDS (O, N, F, and B) mappings).


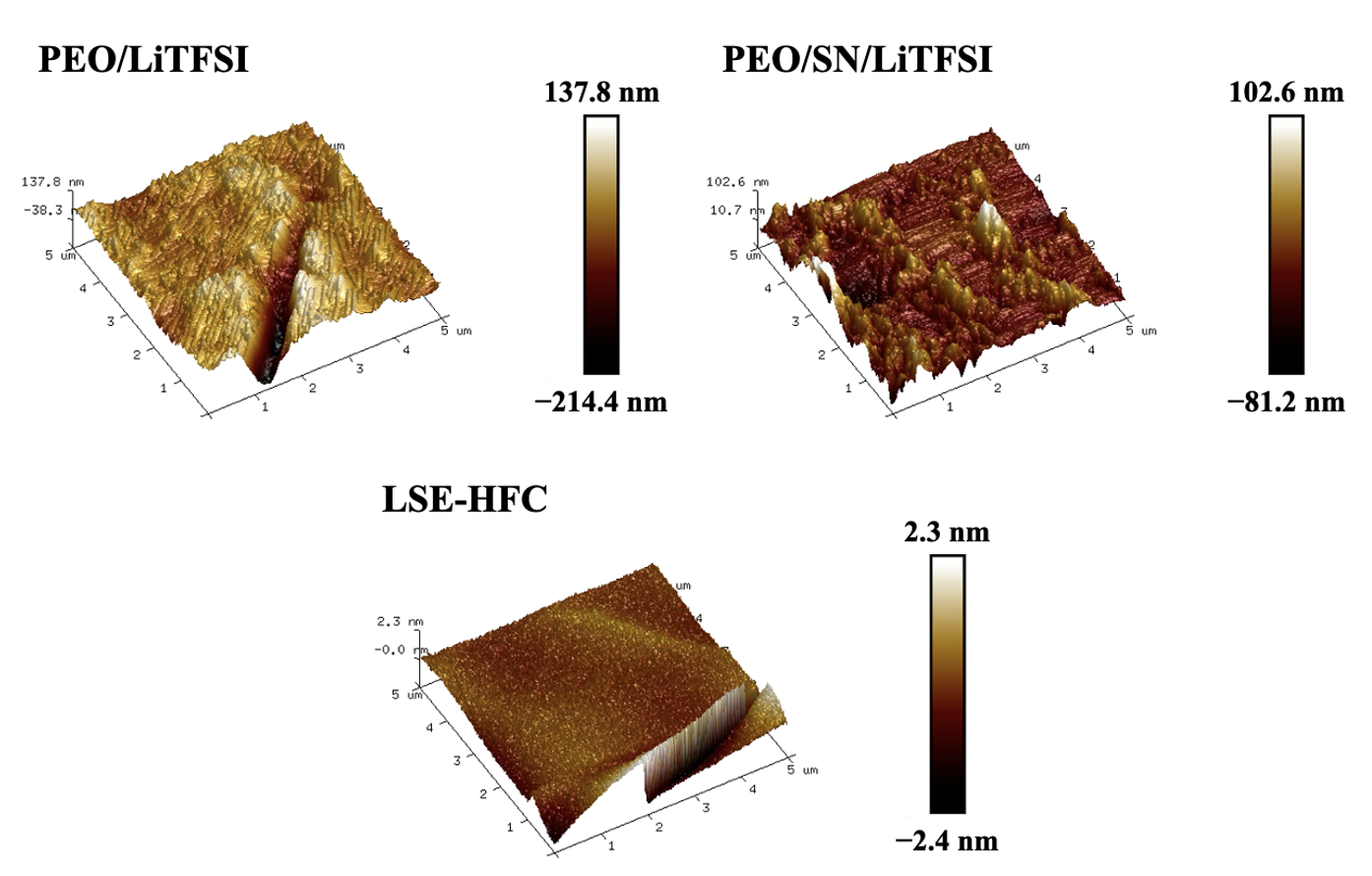


**Figure S10.** AFM images of PEO/LiTFSI, PEO/SN/LiTFSI, and LSE-HFC.


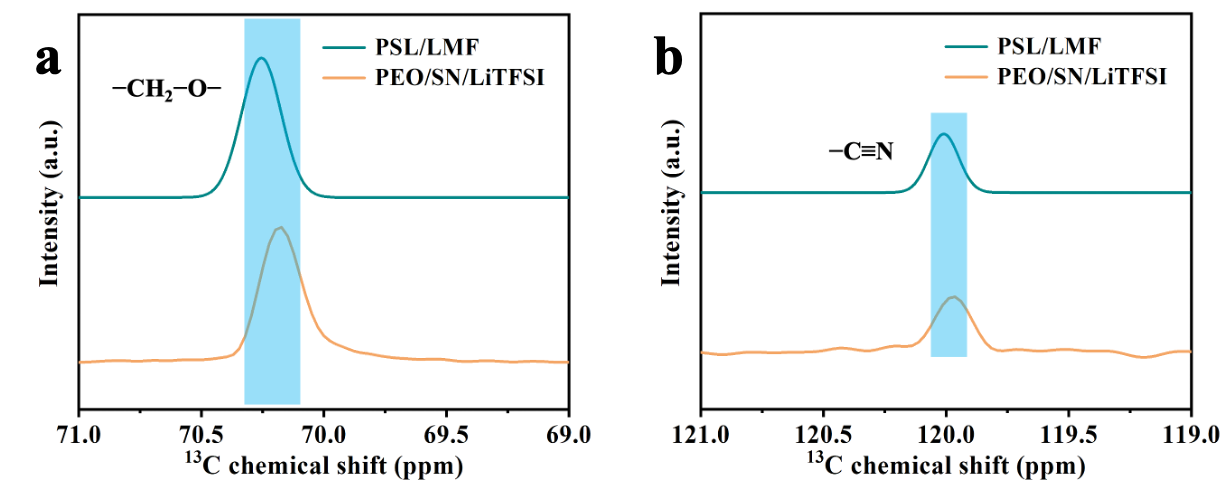


**Figure S11.** (a) and (b) ^13^C NMR spectra of PSL/LMF and PEO/SN/LiTFSI.


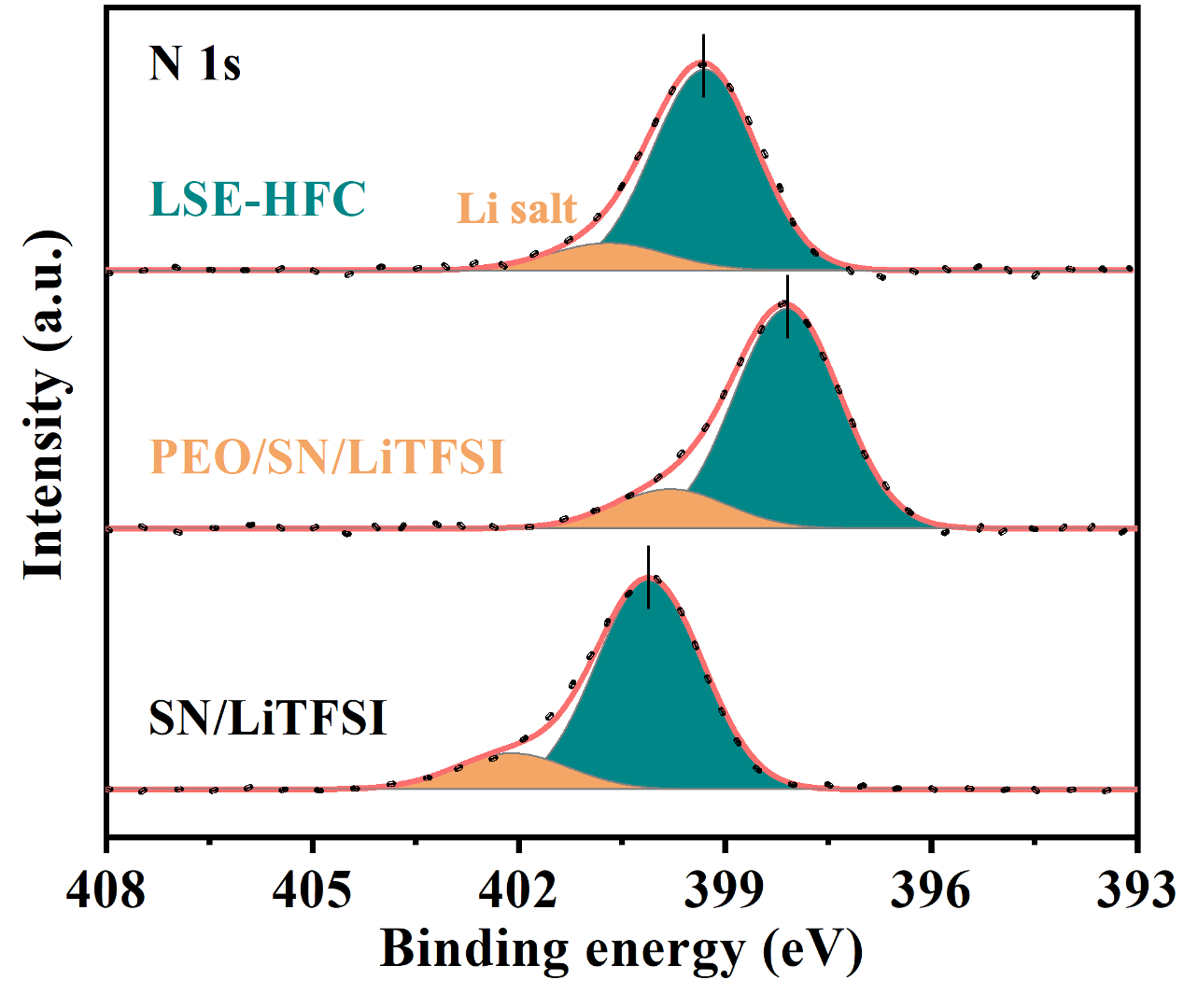


**Figure S12.** N 1s XPS spectra of SN/LiTFSI, PEO/SN/LiTFSI, and LSE-HFC.


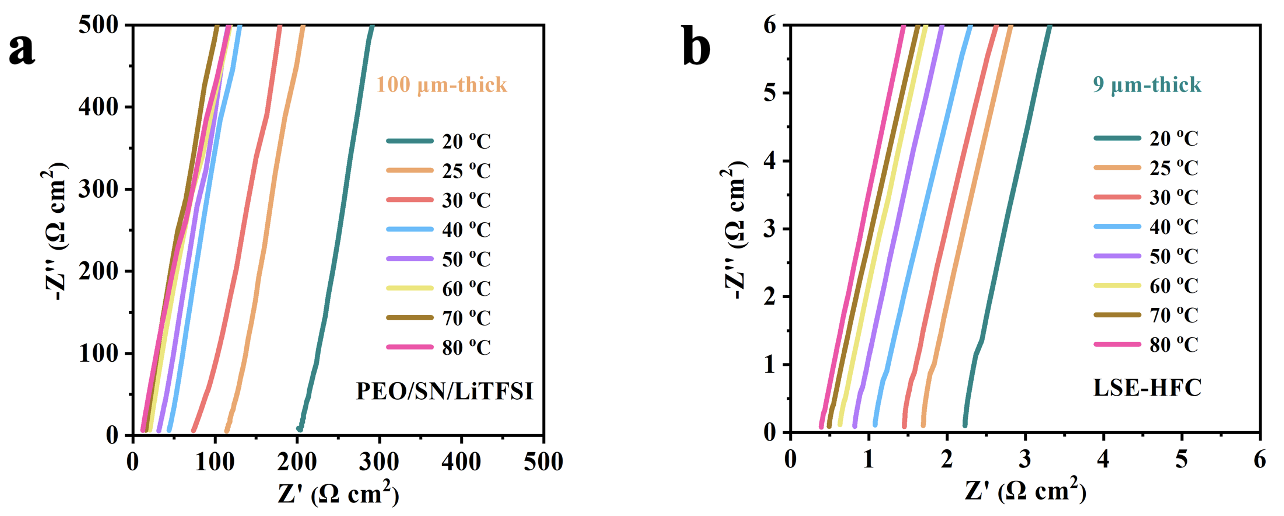


**Figure S13.** The Nyquist plots of (a) PEO/SN/LiTFSI and (b) LSE-HFC (20−80 ^o^C).


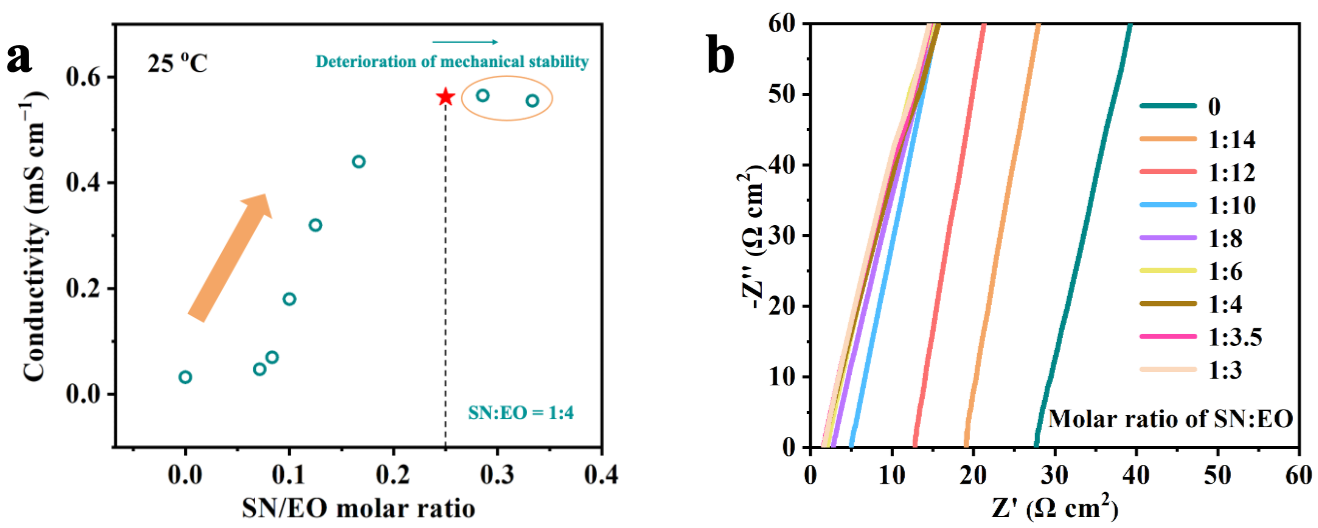


**Figure S14.** (a) Variation of ionic conductivity of LSEs with different SN contents at 25 ^o^C and (b) the corresponding EIS data.


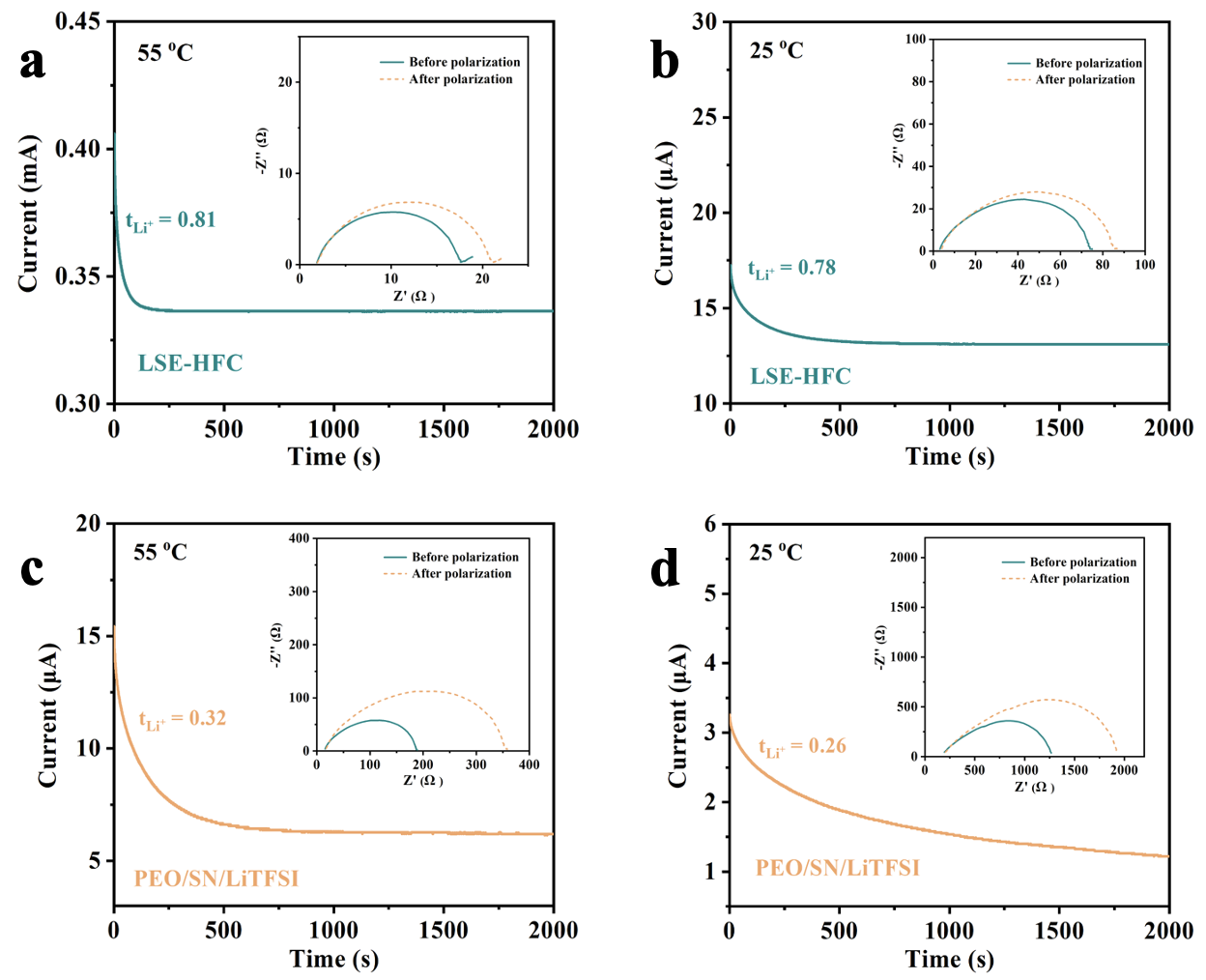


**Figure S15.** The chronoamperometry curves of (a) and (b) LSE-HFC at 55 ^o^C and 25 ^o^C, respectively, (c) and (d) PEO/SN/LiTFSI at 55 ^o^C and 25 ^o^C, respectively (inset: AC impedance curves of the corresponding cells before and after polarization).


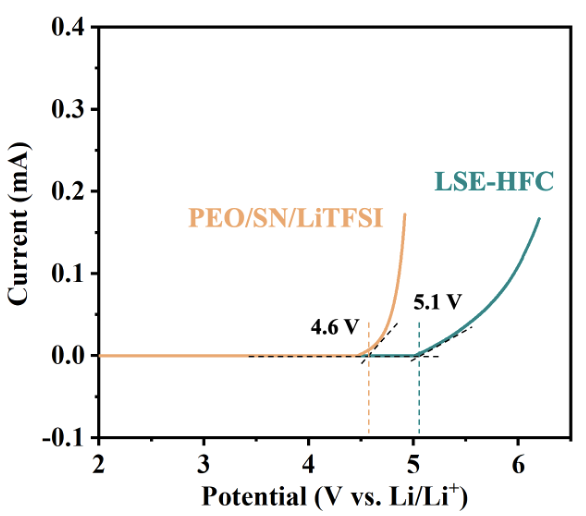


**Figure S16.** LSV curves of PEO/SN/LiTFSI and LSE-HFC.


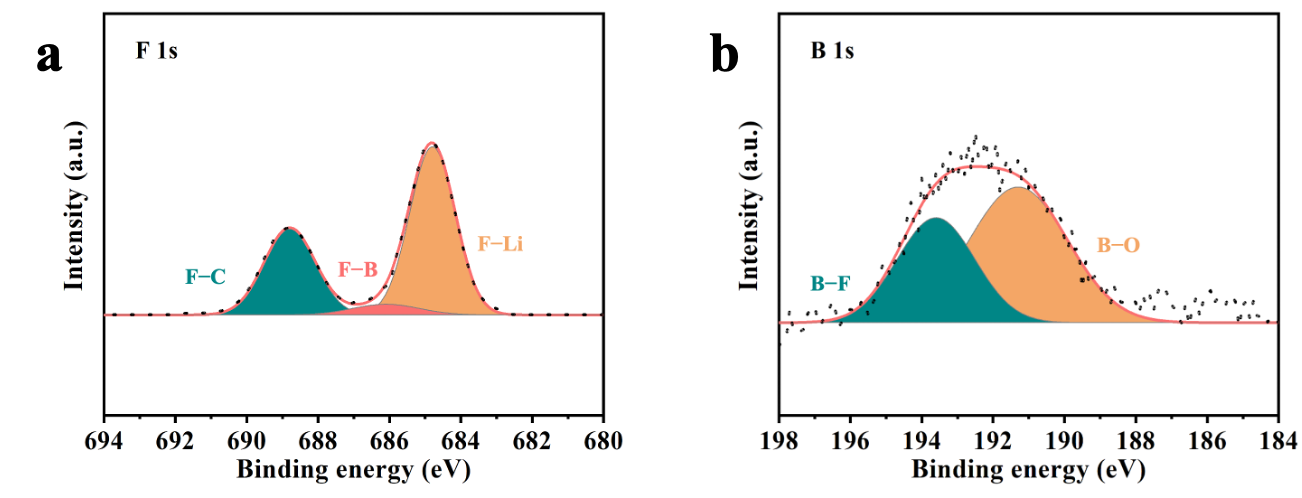


**Figure S17.** (a) F 1s and (b) B 1s XPS spectra of the SEI film formed on cycled Li electrode in Li/LSE-HFC/Li.


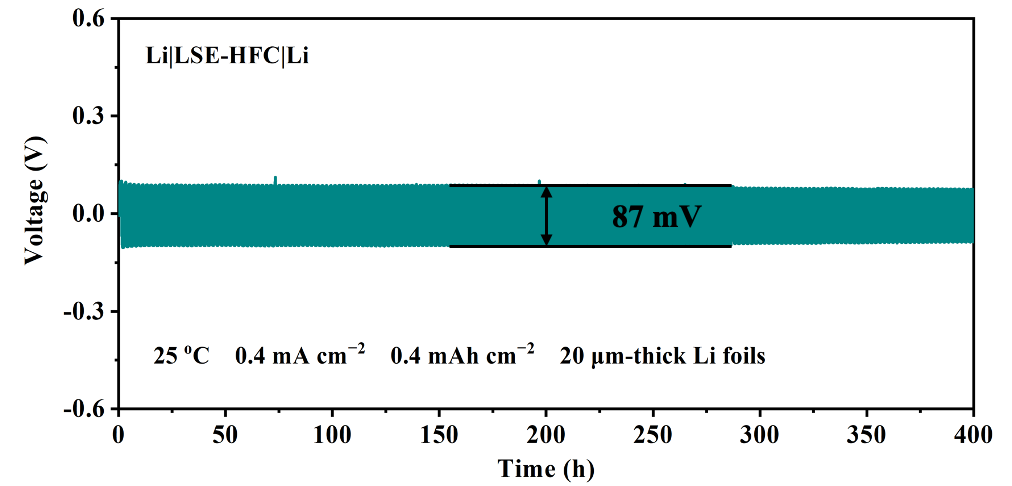


**Figure S18.** Long-term cycling of Li|LSE-HFC|Li cell under 25 ^o^C at 0.4 mA cm^−2^ and 0.4 mAh cm^−2^.


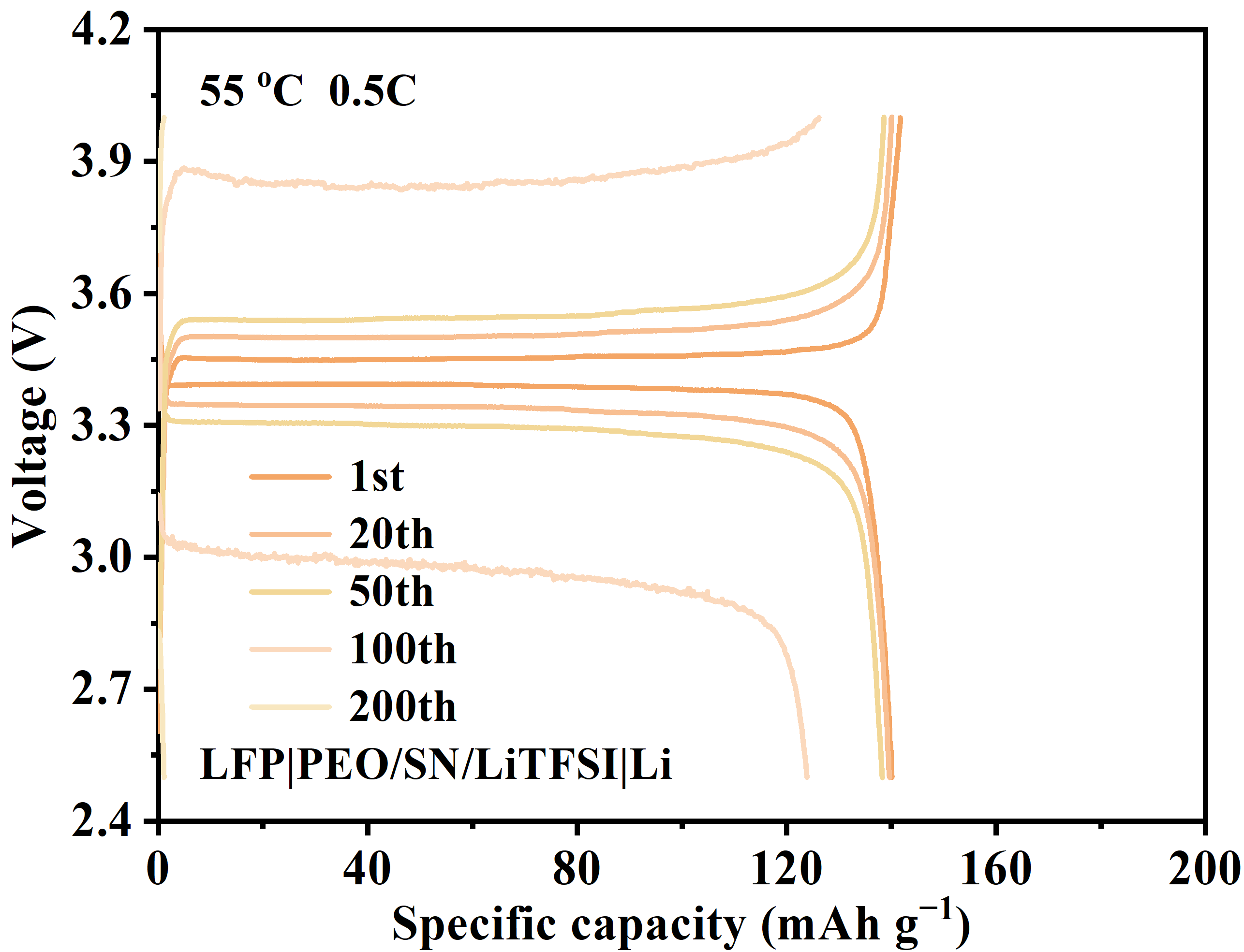


**Figure S19.** Voltage profiles of LFP|PEO/SN/LiTFSI|Li cell at different cycles under 55 ^o^C and 0.5C.


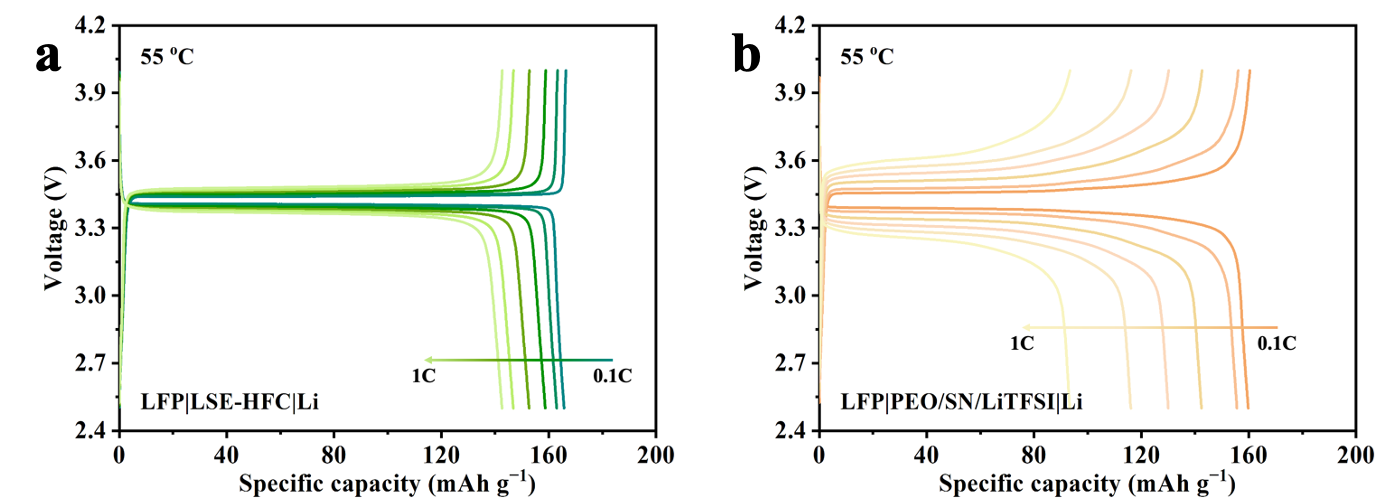


**Figure S20.** Voltage profiles of (a) LFP|LSE-HFC|Li cell and (b) LFP|PEO/SN/LiTFSI|Li cell at different rates under 55 ^o^C.


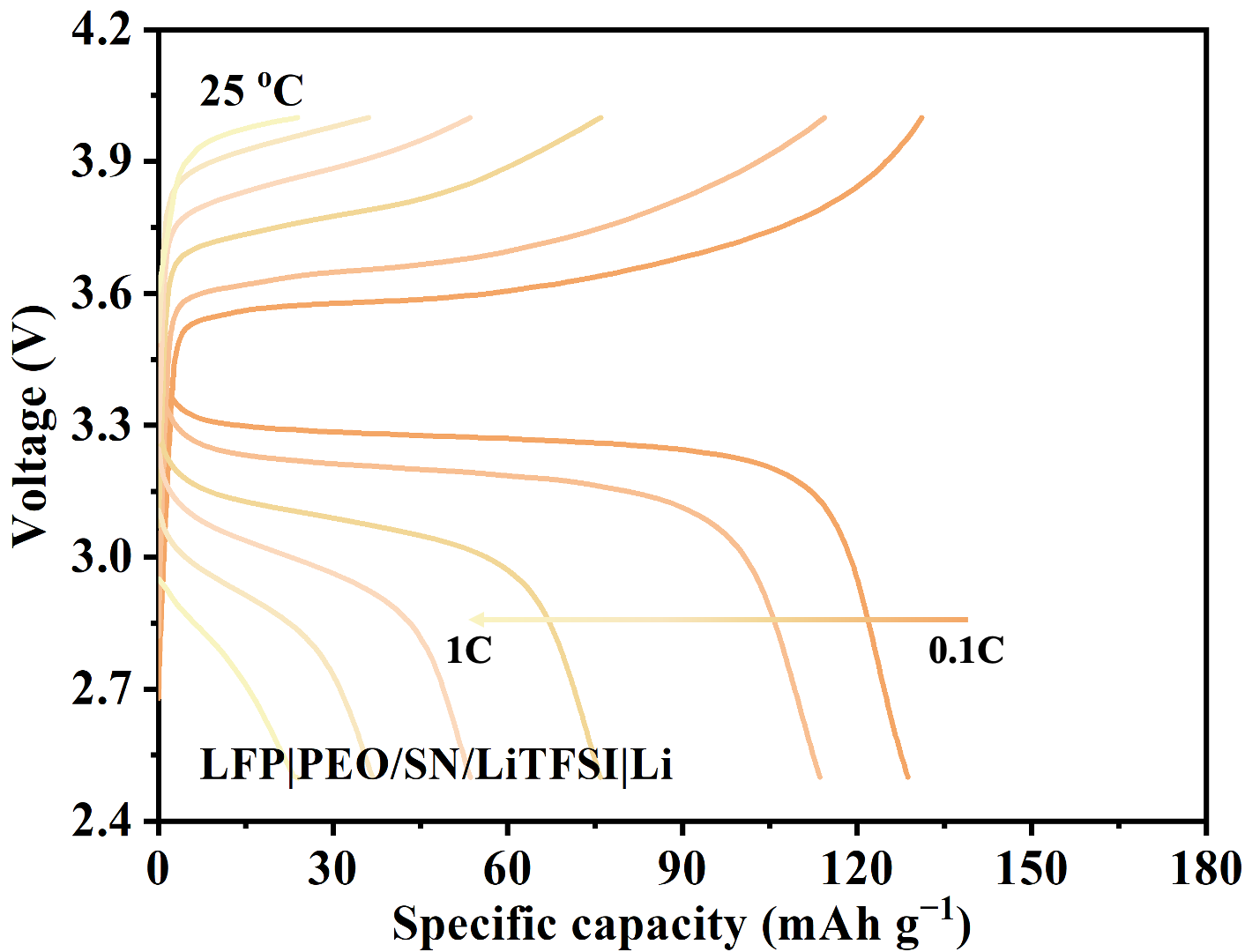


**Figure S21.** Voltage profiles of LFP|PEO/SN/LiTFSI|Li cell at different rates under 25 ^o^C.


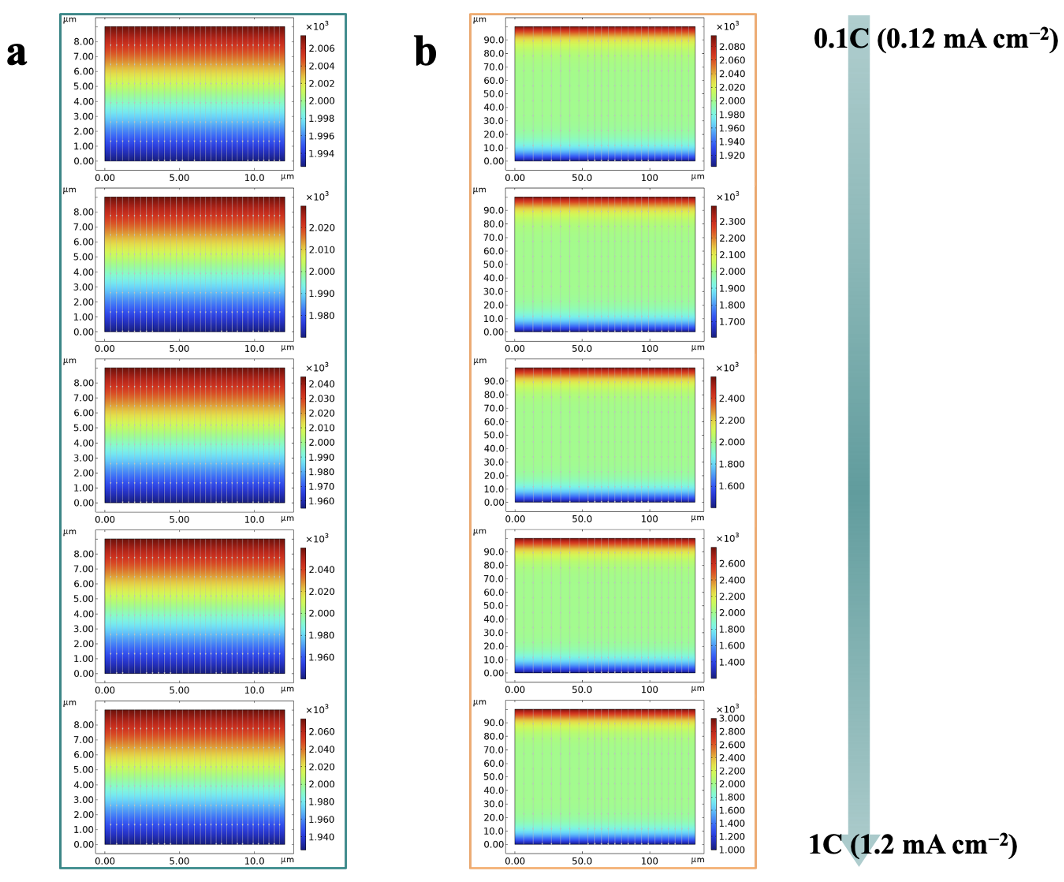


**Figure S22.** Simulation of Li-ion distribution in the (a) LSE-HFC and (b) PEO/SN/LiTFSI at different current densities.


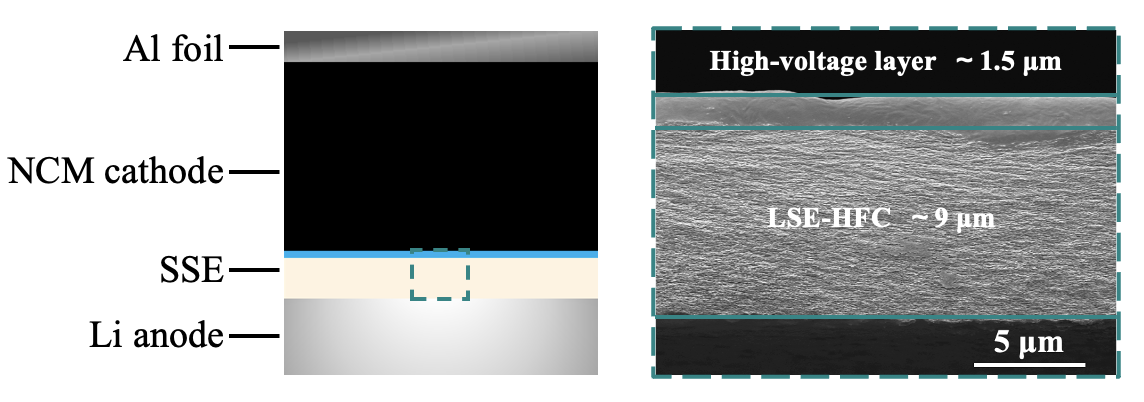


**Figure S23.** Schematic of NCM cell assembled with LSE-HFC@HVL and cross-sectional SEM image of LSE-HFC@HVL.


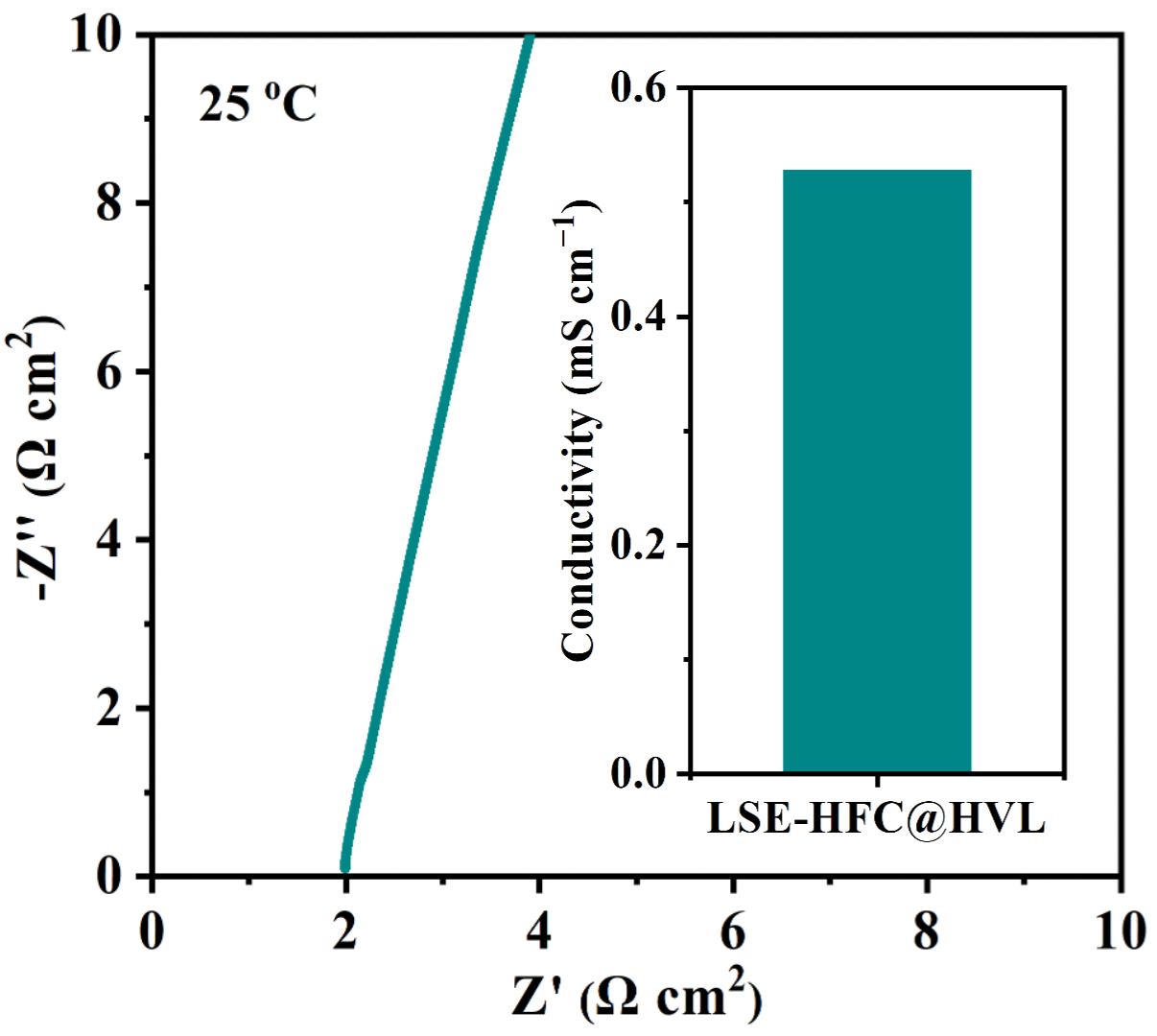


**Figure S24.** The ionic conductivity of LSE-HFC@HVL at 25 ^o^C.


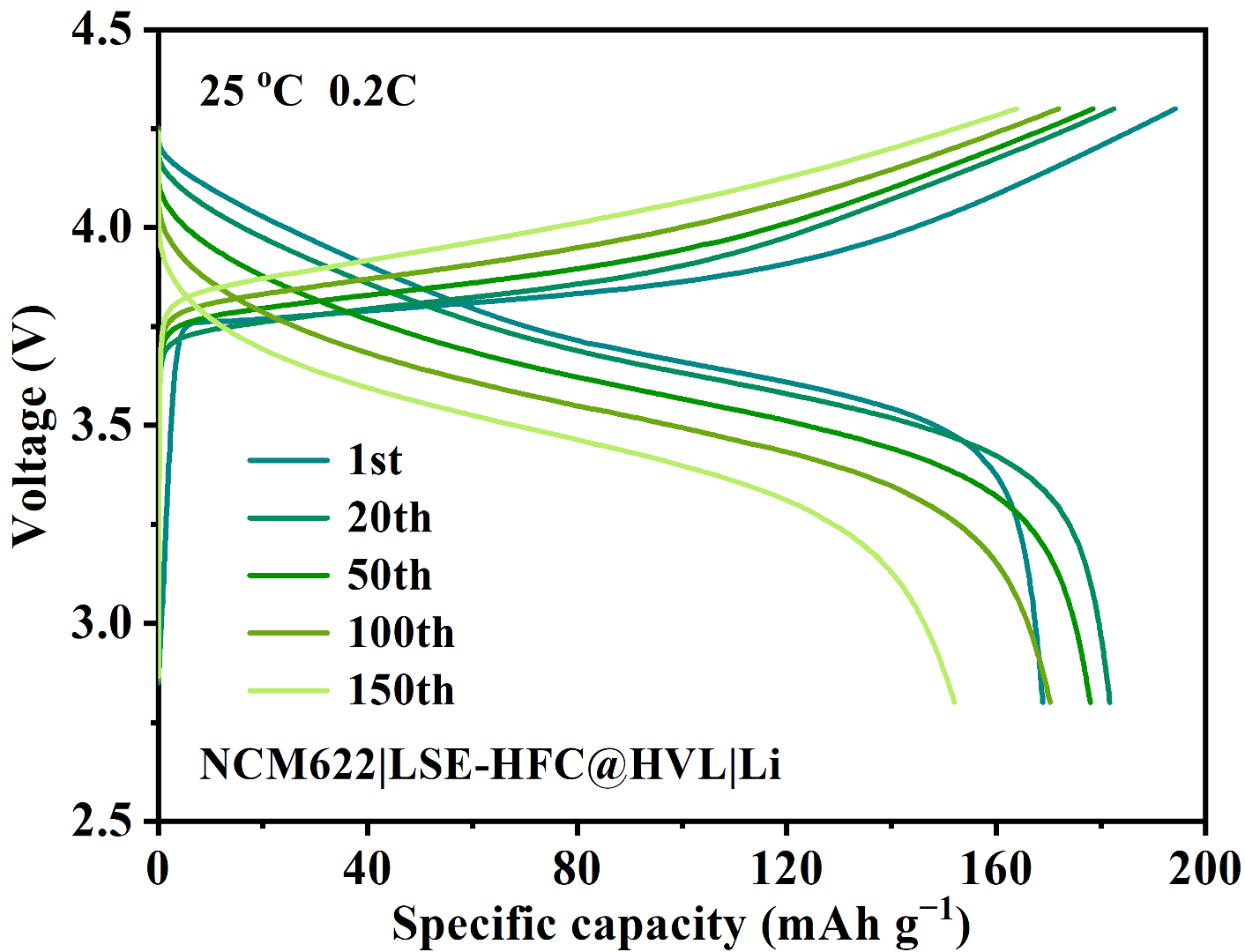


**Figure S25.** Voltage profiles of NCM622|LSE-HFC@HVL|Li cell at different cycles under 25 ^o^C and 0.2C.


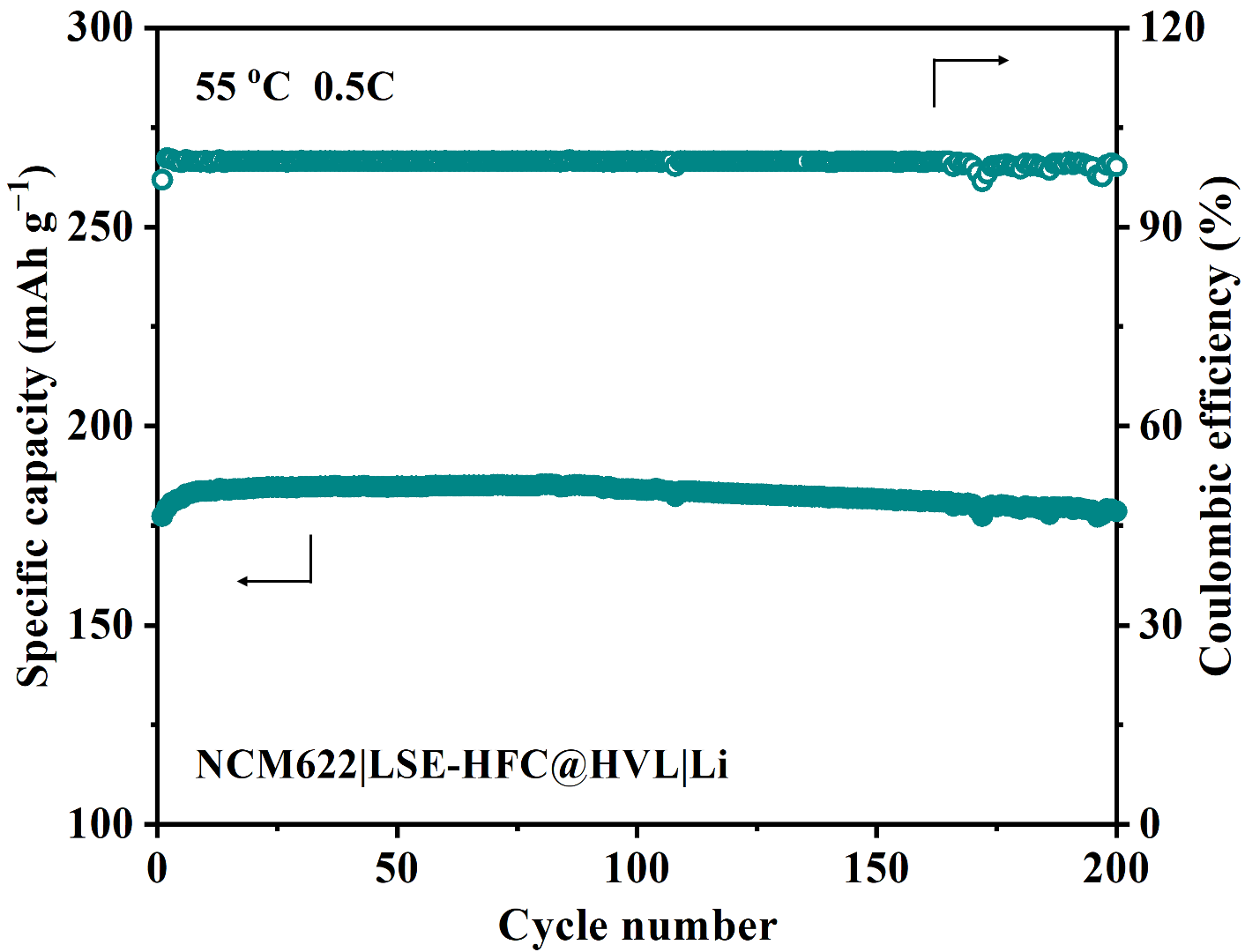


**Figure S26.** Cycling performance at 55 ^o^C and 0.5C of NCM622|LSE-HFC@HVL|Li cell.


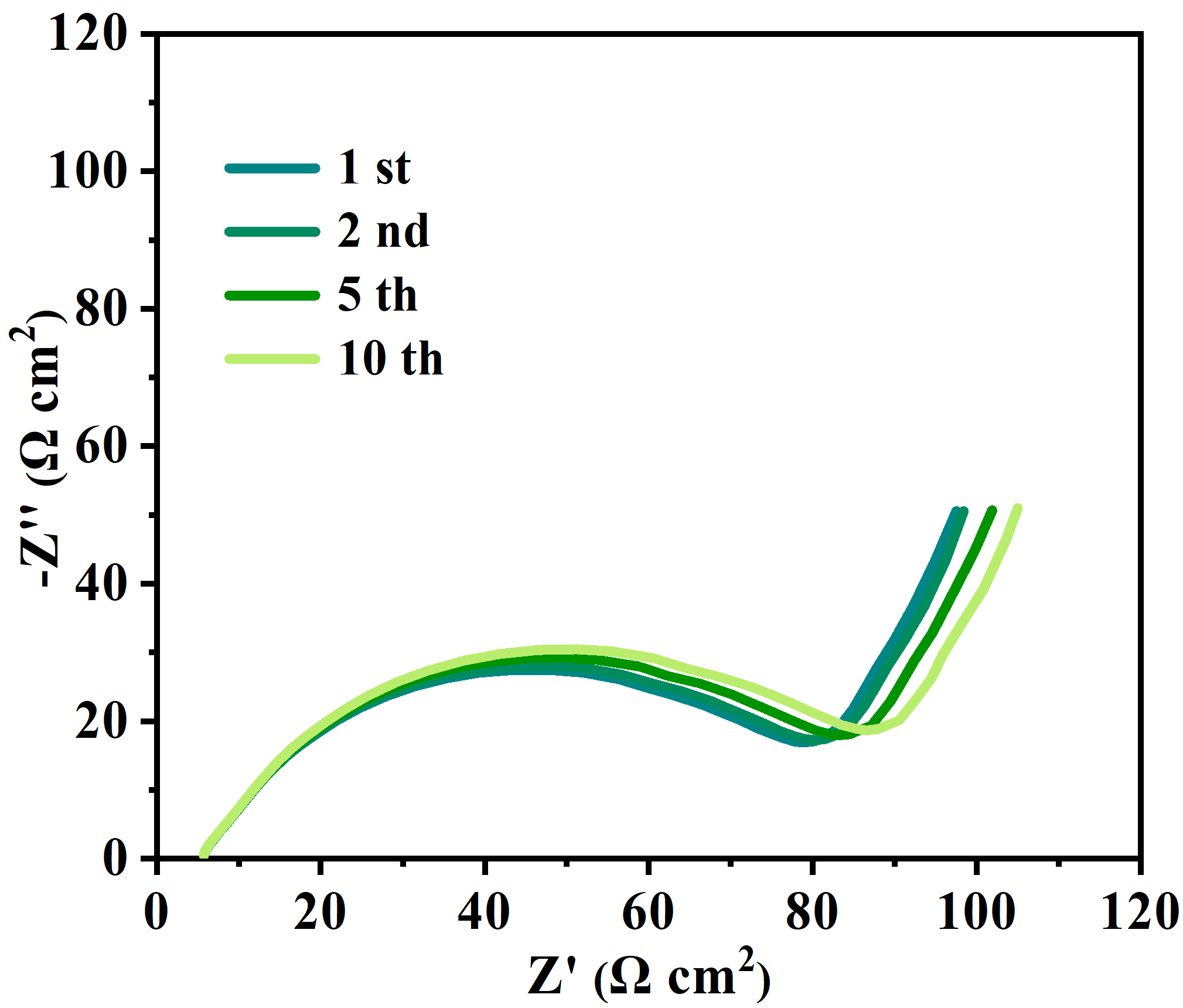


**Figure S27.** Nyquist plots of NCM622|LSE-HFC@HVL|Li cell at 25 ^o^C and 0.2C for ten cycles at 0% SOC.


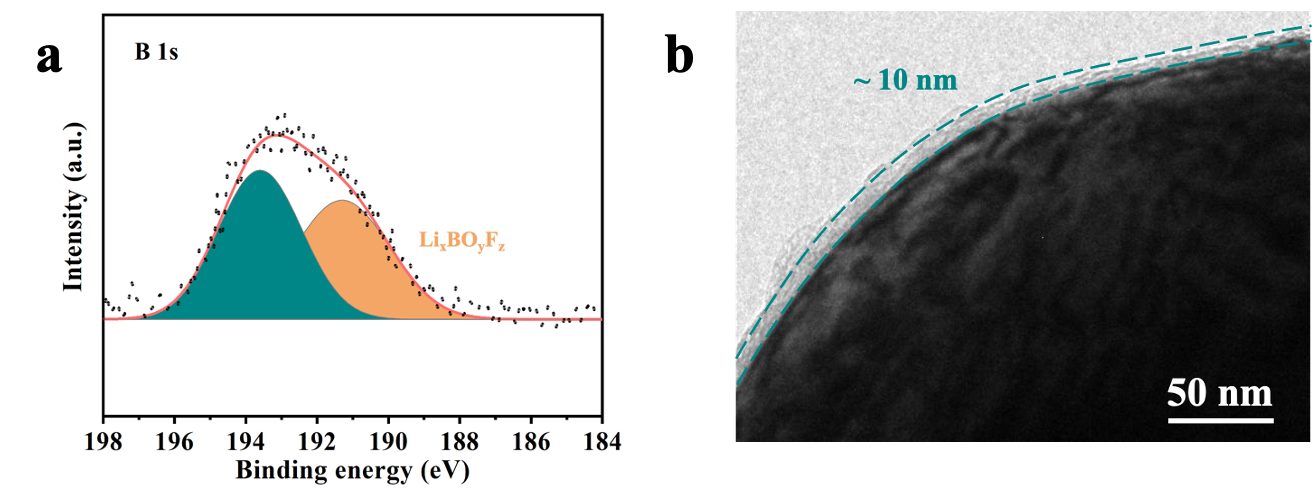


**Figure S28.** (a) B 1s XPS spectra and (b) TEM image of the NCM622 cathode with LSE-HFC@HVL after 10 cycles.


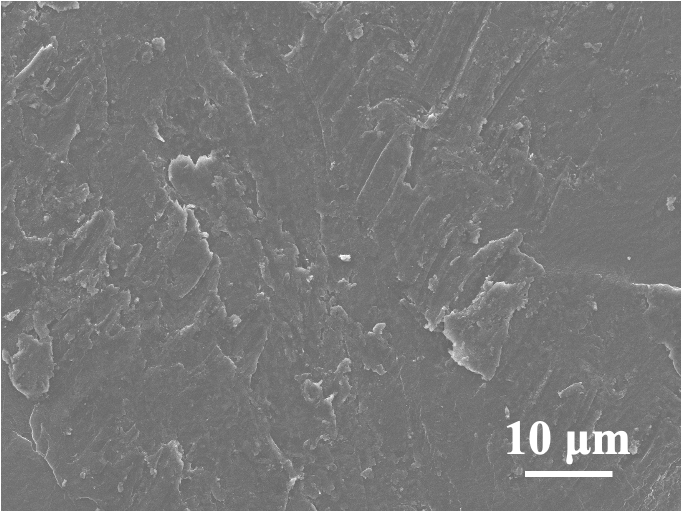


**Figure S29.** The surface SEM image of Li anode with LSE-HFC@HVL after 10 cycles.

**Table S1.** Summary of the performances of recently reported SSEs.

| Solid-state electrolyte | | Thickness (µm) | Li-ion conductivity at RT (S cm^−1^) | Young’s modulus (GPa) | Reference |
| --- | --- | --- | --- | --- | --- |
| LLTO/Vr | 15.0 | | 8.22 × 10^−5^ | 1.24 | 1 |
| PAN/PEO/UiO-66 | 100.0 | | 2.53 × 10^−5^ | 0.82 | 2 |
| PVDF-HFP/PIL/TEOS | 17.0 | | 3.55 × 10^−4^ | 0.72 | 3 |
| PVDF-HFP/UiO-66-SO_3_Li | 10.0 | | 1.00 × 10^−4^ | 0.52 | 4 |
| PEO/PE | 7.5 | | 7.36 × 10^−6^ | 0.45 | 5 |
| PEO/PI | 8.6 | | 4.60 × 10^−5^ | 0.85 | 6 |
| PEO/SiO_2_ | 600.0 | | 2.28 × 10^−4^ | 0.43 | 7 |
| PEGDA/MPS/BN | 80.0 | | 5.15 × 10^−5^ | 0.026 | 8 |
| PEGM/BC | 10.0 | | 2.64 × 10^−4^ | 0.74 | 9 |
| PME/Dual-Li | 70.0 | | 2.21 × 10^−4^ | 3.30 | 10 |
| LSE-HFC | 9.0 | | 4.38 × 10^−4^ | 1.38 | This work |

**Table S2.** Component and weight of ASSLMB for the calculation of gravimetric energy density.

| Component | Cathode | Al foil | Li anode | SSEs |
| --- | --- | --- | --- | --- |
| Weight (mg cm^−2^) | 5.0 (LFP at 55 ^o^C) | 2.7 | 1.02 | 0.648 (LSE-HFC) |
|  | 8.75 (LFP at 25 ^o^C) |  |  |  |
|  | 8.75 (NCM at 25 ^o^C) |  |  | 0.893 (LSE-HFC@HVL) |

Energy density for ASSLMB was calculated by the following Faraday formula:

$$\text{E}_{\text{g}}\text{ = }\frac{\text{V∙}\text{m}_{\text{a}}\text{∙C}}{\text{∑}\text{W}_{\text{i}}}$$

Where *E_g_*, *V*, *m_a_*, *C*, and *W_i_* represent gravimetric energy density (Wh kg^−1^), average cell operating voltage (V), active material loading (mg cm^−2^), active material capacity (mAh g^−1^), and weight of individual cell components (mg cm^−2^), respectively.

The average cell operating voltages of LFP and NCM622 are 3.4 and 3.8 V, respectively. The active material capacities of LFP and NCM622 are 170 and 190 mAh g^−1^, respectively. Since two side coating of electrode materials on collector, the weight of collector should be divided by two. Thus, the gravimetric energy densities are 288.4, 343.8, and 420.7 Wh kg^−1^ for LFP|LSE-HFC|Li cell at 55 ^o^C, 25 ^o^C, and NCM622|LSE-HFC@HVL|Li cell at 25 ^o^C, respectively.

**Table S3.** Summary of the performances of recently reported ASSLMBs.

| Battery system | Energy density (Wh kg^−1^) | Power density  (W kg^−1^) | Cycle number | Operation temperature (^o^C) | Reference |
| --- | --- | --- | --- | --- | --- |
| LFP/Li | 164.2 | 82.1 | 200 | 25 | 4 |
|  | 216.0 | 21.6 | 50 | 60 | 5 |
|  | 91.0 | 18.2 | 300 | 60 | 11 |
|  | 48.5 | 24.3 | 420 | 30 | 12 |
|  | 437.1 | 43.7 | 185 | 25 | 13 |
|  | 288.4 | 144.2 | 600 | 55 | This work |
|  | 343.8 | 68.8 | 300 | 25 | This work |
| NCM/Li | 240.6 | 120.3 | 100 | 60 | 2 |
|  | 168.8 | 33.8 | 150 | 25 | 4 |
|  | 486.0 | 48.6 | 150 | 50 | 14 |
|  | 419.6 | 42.0 | 35 | 30 | 15 |
|  | 84.9 | 8.5 | 100 | 25 | 16 |
|  | 420.7 | 84.1 | 150 | 25 | This work |
| LCO/Li | 193.1 | 19.3 | 100 | 60 | 17 |
|  | 182.0 | 9.1 | 100 | 25 | 18 |
|  | 52.4 | 21.0 | 430 | 60 | 19 |
| LNMO/Li | 260.2 | 26.0 | 100 | 25 | 20 |
| LTO/Li | 72.0 | 36.0 | 125 | 50 | 21 |

**References**

[1] R. Lv, W. Kou, S. Guo, W. Wu, Y. Zhang, Y. Wang, J. Wang, *Angew. Chem., Int. Ed.* **2022**, *61*, e202114220.

[2] Z. Li, S. Wang, J. Shi, Y. Liu, S. Zheng, H. Zou, Y. Chen, W. Kuang, K. Ding, L. Chen, Y. Lan, Y. Cai, Q. Zheng, *Energy Storage Mater.* **2022**, *47*, 262.

[3] M. Yao, Q. Ruan, Y. Wang, L. Du, Q. Li, L. Xu, R. Wang, H. Zhang, *Adv. Funct. Mater.* **2023**, *33*, 2213702.

[4] M. Yao, Q. Ruan, S. Pan, H. Zhang, S. Zhang, *Adv. Energy Mater*. **2023**, *34*, 2203640.

[5] J. Wu, Z. Rao, Z. Cheng, L. Yuan, Z. Li, Y. Huang, *Adv. Energy Mater.* **2019**, *9*, 201902767.

[6] J. Wan, J. Xie, X. Kong, Z. Liu, K. Liu, F. Shi, A. Pei, H. Chen, W. Chen, J. Chen, X. Zhang, L. Zong, J. Wang, L. Chen, J. Qin, Y. Cui, *Nat. Nanotechnol.* **2019**, *14*, 705.

[7] D. Lin, P. Y. Yuen, Y. Liu, W. Liu, N. Liu, R. H. Dauskardt, Y. Cui, *Adv. Mater.* **2018**, *30*, 201802661.

[8] H. An, Q. Liu, J. An, S. Liang, X. Wang, Z. Xu, Y. Tong, H. Huo, N. Sun, Y. Wang, Y. Shi, J. Wang, *Energy Storage Mater.* **2021**, *43*, 358.

[9] M. Zhou, R. Liu, D. Jia, Y. Cui, Q. Liu, S. Liu, D. Wu, *Adv. Mater.* **2021**, *33*, 2100943.

[10] H. Li, Y. Du, X. Wu, J. Xie, F. Lian, *Adv. Funct. Mater.* **2021**, *31*, 2103049.

[11] W. Sun, J. Zhang, M. Xie, D. Lu, Z. Zhao, Y. Li, Z. Cheng, S. Zhang, H. Chen, *Nano Lett.* **2020**, *20*, 8120.

[12] H. Zhou, Y. Ou, S. Yan, J. Xie, P. Zhou, L. Wan, Z. Xu, F. Liu, W. Zhang, Y. Xia, K. Liu, *Angew. Chem., Int. Ed.* **2023**, *62*, e202306948.

[13] S. Chen, J. Zhang, L. Nie, X. Hu, Y. Huang, Y. Yu, W. Liu, *Adv. Mater.* **2021**, *33*, 2002325.

[14] F. He, W. Tang, X. Zhang, L. Deng, J. Luo, *Adv. Mater.* **2021**, *33*, 2105329.

[15] Y. Ouyang, W. Gong, Q. Zhang, *Adv. Mater.* **2023**, *35*, 2304685.

[16] S. Wang, Q. Sun, Q. Zhang, C. Li, C. Xu, Y. Ma, X. Shi, H. Zhang, D. Song, L. Zhang, *Adv. Energy Mater.* **2023**, *13*, 2204036.

[17] C. Wang, T. Wang, L. Wang, Z. Hu, Z. Cui, J. Li, S. Dong, X. Zhou, G. Cui, *Adv. Sci.* **2019**, *6*, 1901036.

[18] F. Han, J. Yue, C. Chen, N. Zhao, X. Fan, Z. Ma, T. Gao, F. Wang, X. Guo, C. Wang, *Joule* **2018**, *2*, 497.

[19] W. Xiao, H. Xu, M. Xuan, Z. Wu, Y. Zhang, X. Zhang, S. Zhang, Y. Shen, G. Shao, *J. Energy Chem.* **2020**, *53*, 147.

[20] D. Lee, Z. Cui, J. B. Goodenough, A. Manthiram, *Small* **2024**, *20*, 2306053.

[21] K. Pan, L. Zhang, W. Qian, X. Wu, K. Dong, H. Zhang, S. Zhang, *Adv. Mater.* **2020**, *32*, 2000399.
